# AT-RvD1 liposomes effectively accumulate in spinal cord and reprogram microglia via ANGPTL4-SUCLG1-propionate axis for treating neuropathic pain

**DOI:** 10.1016/j.mtbio.2026.103726

**Published:** 2026-10-05

**Authors:** Kaili Wu, Chao Gao, Shunli Fu, Yan Li, Zhaohui Li, Xinyi Han, Yun Zhang, Yingqi Zhang, Yihao Wang

**Affiliations:** a Department of Pain Management, The Affiliated Hospital of Qingdao University, Qingdao University, Qingdao, 266000, China; b Department of Anesthesiology, The Affiliated Hospital of Qingdao University, Qingdao University, Qingdao, 266000, China; c Tumor Immunology and Cytotherapy of Medical Research Center, Shandong Provincial Key Laboratory of Clinical Research for Pancreatic Diseases, the Affiliated Hospital of Qingdao University, Qingdao University, Qingdao, 266000, China; d Department of Obstetrics, The Affiliated Hospital of Qingdao University, Qingdao University, Qingdao, 266000, China; e Department of Spine Surgery, The Affiliated Hospital of Qingdao University, Qingdao University, Qingdao, 266000, China

**Keywords:** Neuropathic pain, AT-RvD1, ANGPTL4, Microglial inflammatory responses, MG1 targeted liposome nanoparticle

## Abstract

Neuropathic pain (NP) remains a major clinical challenge as current medications are ineffective in treating the underlying cause, and the clinical use of these drugs also has adverse reactions such as addiction. Therefore, specialized pro-resolving mediators (SPMs) may represent a promising therapeutic strategy. Microglia-mediated neuroinflammation is central to NP pathogenesis, yet feasible strategies for precise modulation of microglial inflammatory responses remain limited. Here, we reveal that aspirin-triggered resolvin D1 (AT-RvD1) promotes a shift of microglia toward an anti-inflammatory and pro-repair state via a novel ANGPTL4-SUCLG1-propionate axis, consequently attenuating neuroinflammation and pain hypersensitivity in mice models with NP. To address invasive nature of intrathecal injection limitation in NP clinical treatment, an MG1 peptide-modified liposomal delivery system encapsulating AT-RvD1 (AT-RvD1@Lipo-MG1) is developed. Intravenous administration of this nanoplatform enables effective and targeted delivery of AT-RvD1 to spinal microglia, with robust analgesic and anti-inflammatory effects and favorable biosafety in vivo. This study elucidates a novel mechanism through which AT-RvD1 modulates microglial inflammatory responses and proposes a highly translatable nanotherapeutic strategy for the treatment of NP.

## Graphical abstract

Graphical abstract:AT-RvD1 alleviates neuropathic pain by regulating microglial inflammatory responses via the ANGPTL4/SUCLG1/propionate axis. It suppresses ANGPTL4, promotes SUCLG1 degradation, and increases spinal propionate levels, shifting microglia from a pro-inflammatory to an anti-inflammatory state. MG1-modified liposomes enable targeted delivery to spinal microglia, overcoming poor druggability and enhancing therapeutic efficacy.

**Figure:**
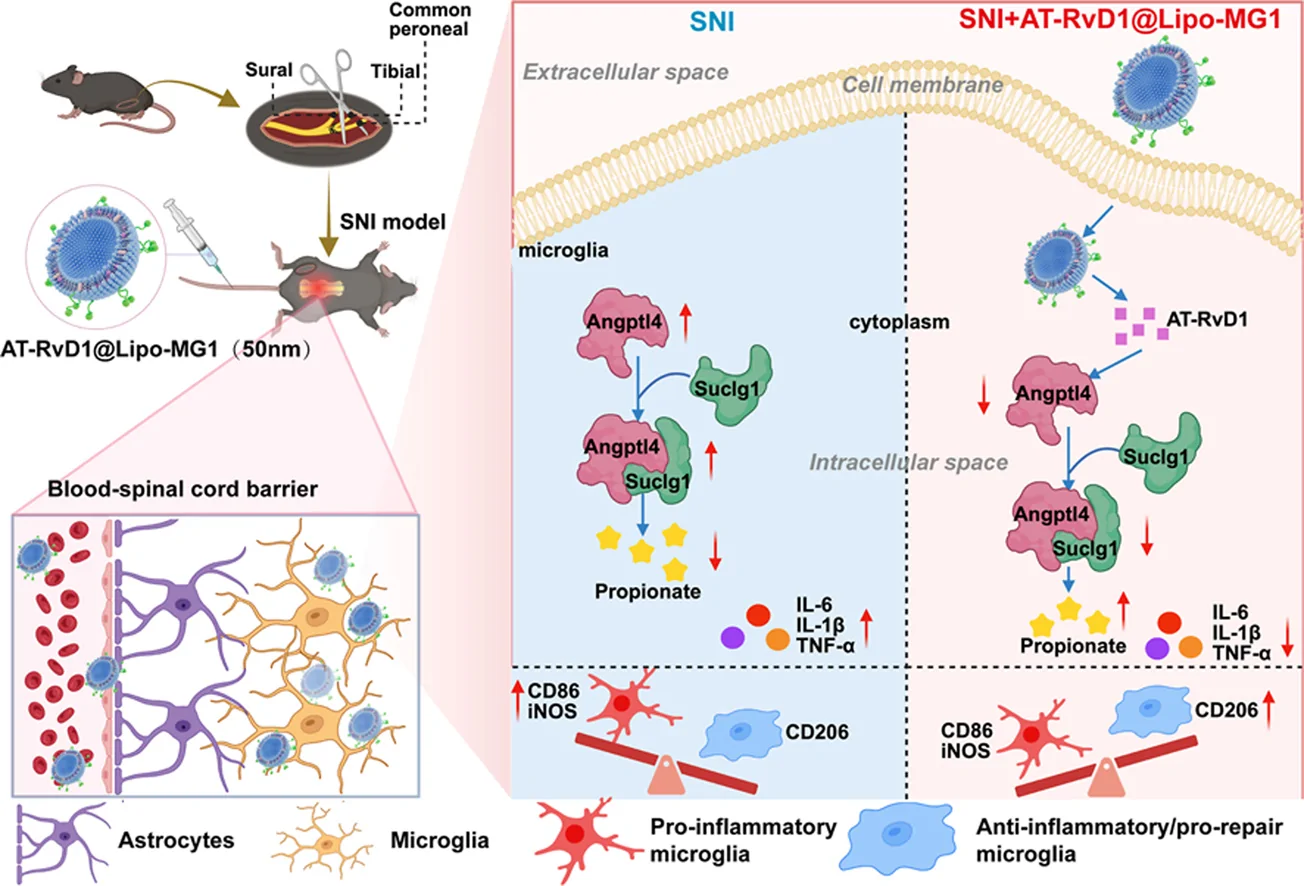

## Abbreviations

SPMs: specialized pro-resolving mediators
AT-RvD1: aspirin-triggered resolvin D1
AT-RvD1@Lipo-MG1: MG1 peptide-modified liposomal delivery system encapsulating AT-RvD1
SNRIs: serotonin-norepinephrine reuptake inhibitors
EI: electron ionization
DIA: data-independent acquisition
FDR: false discovery rate
IP-MS: immunoprecipitation combined with mass spectrometry
PWT: paw withdrawal threshold
MFI: mean fluorescence intensity
GC-MS: Gas Chromatography-Mass Spectrometry
Co-IP: co-immunoprecipitation
CHX: cycloheximide
CQ: chloroquine
RhB: Rhodamine B
CNS: central nervous system
EPA: eicosapentaenoic acid
DHA: docosahexaenoic acid
COX-2: cyclooxygenase-2

## 1 Introduction

Neuropathic pain (NP), defined as a chronic and refractory pain condition, arises from lesions or diseases that affect the somatosensory nervous system, and is typically manifested by spontaneous pain, hyperalgesia, allodynia, and abnormal sensations in clinical [1,2]. In a large epidemiological study of middle-aged people in the UK, the incidence of chronic pain was as high as 51.1%, among which the prevalence of NP was 9.2%, seriously affecting the function and quality of patients’ life and increasing the global economic burden [3]. For peripheral and central neuropathic pain , first-line pharmacological treatments currently consist of tricyclic antidepressants, gabapentinoids, and serotonin-norepinephrine reuptake inhibitors (SNRIs); and tramadol and topical treatments (high-concentration capsaicin patches and 5% lidocaine ointment) as second-line treatments; and strong opioids as third-line options [4]. However, these drugs are often constrained by dose-limiting adverse effects, including nausea and vomiting, as well as risks of addiction and abuse, cardiotoxicity, and poor long-term tolerance, resulting in suboptimal therapeutic outcomes. Therefore, it is essential to further explore novel molecular mechanisms and identify safer and more effective therapeutic targets and strategies for NP treatment [5,6].

Neuroinflammation is a core driver of the initiation and maintenance of NP, and aberrant microglial activation is a central pathological node in neuroinflammatory cascades within the nervous system [7,8]. Microglia, serving as the resident immune cells of the central nervous system (CNS), display marked phenotypic plasticity and have traditionally been described in terms of pro-inflammatory (M1-like) and anti-inflammatory/pro-repair (M2-like) states [9]. Among them, M1-like microglia are associated with the secretion of pro-inflammatory mediators, such as tumor necrosis factor (TNF-α), which amplify neuroinflammation and contribute to pain hypersensitivity. In contrast, M2-like microglia facilitate tissue repair and mitigate pain through the release of anti-inflammatory cytokines, including interleukin-10 (IL-10) [10,11]. Accordingly, modulating dysregulated microglial activation and promoting a shift toward an anti-inflammatory and pro-repair state is considered a promising strategy for NP intervention.

Specialized pro-resolving mediators (SPMs), a family of endogenous lipid mediators, are biosynthesized from the omega-3 fatty acids eicosapentaenoic acid (EPA) and docosahexaenoic acid (DHA), which can terminate inflammatory responses and promote the restoration of tissue homeostasis [12]. Resolvins are key members of the SPMs family. Among them, aspirin-triggered resolvin D1 (AT-RvD1) is a 17 R-hydroxylated derivative of DHA, generated via aspirin-acetylated cyclooxygenase-2 (COX-2)-mediated biosynthesis, and belongs to the resolvin D subfamily [13]. These molecules exhibit strong anti-inflammatory and pro-resolving effects during the inflammatory resolution phase [14,15]. Our group has previously conducted systematic investigations on the roles of SPMs in neuroinflammation, and recently found that AT-RvD1 attenuates neuroinflammatory responses in the spinal dorsal horn and alleviates NP [16]. However, the underlying molecular mechanisms underlying the regulatory effects of AT-RvD1 on microglial inflammatory responses and NP progression remain largely unclear.

Angiopoietin-like protein 4 (ANGPTL4), as a key molecule linking lipid metabolism, inflammatory responses, and tissue remodeling, plays critical roles in the pathogenesis of metabolic cardiovascular diseases, tumors, lung injury, neurodegenerative disorders, and pancreatitis [[17], [18], [19]]. Studies have demonstrated that ANGPTL4 enhances NF-κB (p65) expression while suppressing the anti-inflammatory factor SIRT1 in a lipopolysaccharide (LPS)-induced acute lung injury model, thereby aggravating the inflammatory response [20]. In the acute pancreatitis model, the overexpression of ANGPTL4 drives macrophage activation and complement C5a pathway signaling, triggering a “cytokine storm”, which further aggravates tissue inflammatory damage [21]. Recent evidence has also highlighted a pivotal role of ANGPTL4 in the nervous system. It promotes lipid droplet accumulation in microglia, driving metabolic and inflammatory imbalances, thereby exacerbating neuroinflammation and neuronal damage, and contributing to the development and advancement of neurodegenerative diseases such as Alzheimer's disease [22]. Although the role of ANGPTL4 in inflammation regulation has attracted growing attention, no studies have yet investigated whether AT-RvD1 can modulate ANGPTL4 expression to regulate microglial inflammatory responses and neuroinflammation. Here, we hypothesized that AT-RvD1 inhibits ANGPTL4 expression to promote a shift in microglial from a pro-inflammatory toward an anti-inflammatory and pro-repair state, thereby alleviating neuroinflammation and nociceptive hypersensitivity in NP.

Notably, as a polyunsaturated lipid mediator, AT-RvD1 is still limited in its direct application by rapid metabolic degradation and hydrolysis in vivo, as well as insufficient local effective exposure [15,23]. In addition, direct intrathecal administration is highly invasive, making it unsuitable for long-term and repeated treatment [24,25]. Therefore, developing a system that can be administered intravenously and can efficiently deliver to spinal microglial cells is of great significance for its clinical application. Nano-drug delivery systems could encapsulate hydrophilic and lipophilic drugs and offer advantages such as the ability to cross biological barriers, enable controlled drug release, reduced systemic toxicity, prolonged circulation time through PEGylation, passive targeting, and improved drug stability through controlled-release properties [[26], [27], [28], [29], [30], [31], [32], [33]]. Among them, as a classical nanocarrier system, liposome-based drug delivery systems have been successfully applied in clinical practice, including Doxil® and AmBisome®. Furthermore, surface functionalization of liposomes enables precise delivery to pathological sites, thereby significantly enhancing drug accumulation in target tissues while reducing systemic toxicity [34,35]. Hence, liposomes are selected to encapsulate AT-RvD1. MG1 peptide, a ligand that selectively recognizes activated microglia, can specifically bind to microglia within neuroinflammatory regions, thereby providing an ideal tool for the precise targeting of spinal microglia [36]. Based on these premises, this study first delineated the molecular mechanism by which AT-RvD1 regulates microglial inflammatory responses via the ANGPTL4-SUCLG1-propionate axis. Subsequently, we engineered an MG1 peptide-modified AT-RvD1-loaded liposome (AT-RvD1@Lipo-MG1, A@Lipo-MG1) using soy phospholipids and cholesterol as core excipients (Fig. 1). This study provides a minimally invasive, safe, and feasible therapeutic strategy with substantial clinical translation potential for NP management.

**Fig. 1:**
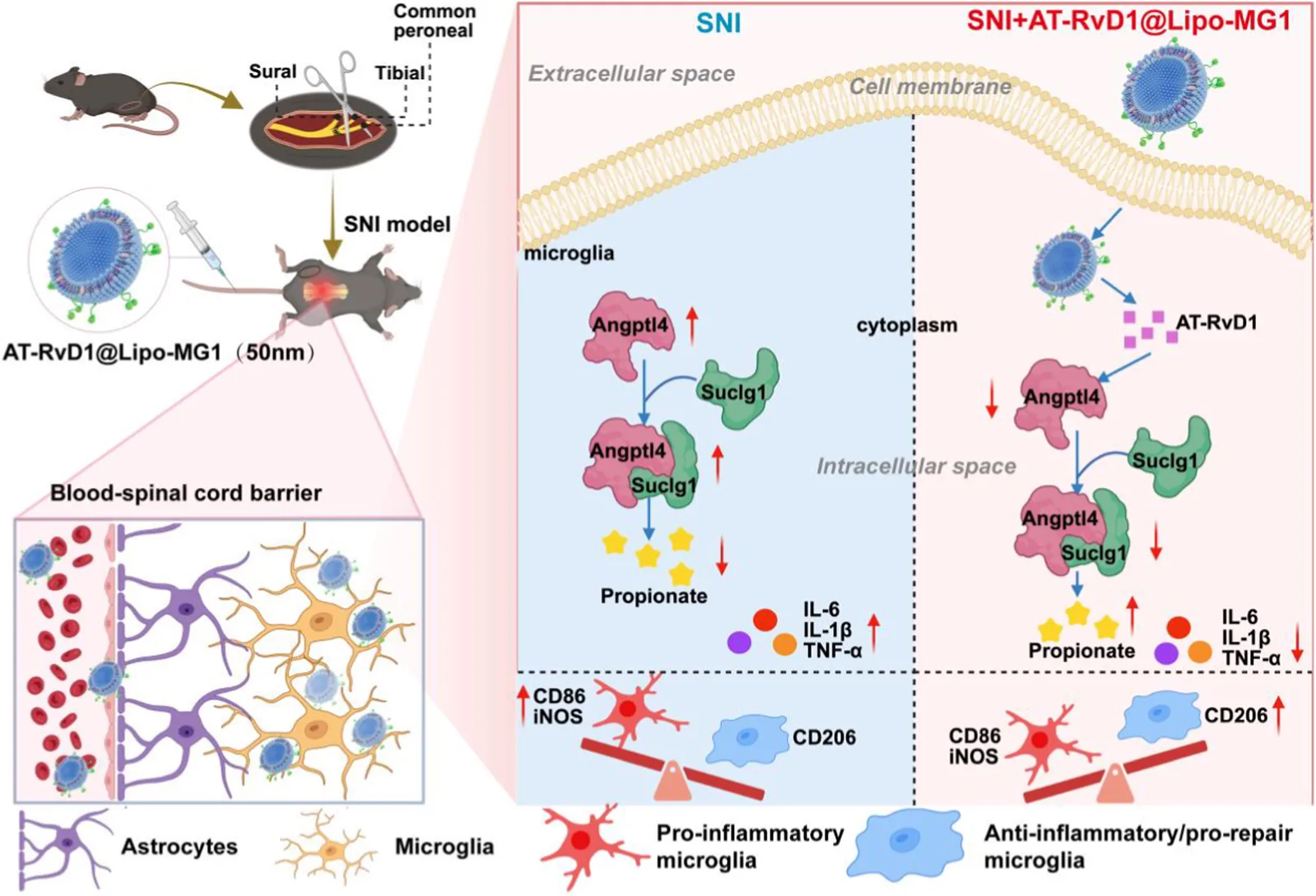
AT-RvD1 alleviates pain hypersensitivity by modulating microglial inflammatory responses through the ANGPTL4/SUCLG1/propionate axis. In the SNI-induced neuropathic pain model, spinal inflammation is markedly activated, accompanied by a significant upregulation of Angptl4 in microglia. Specifically, Angptl4 directly interacts with Suclg1 and inhibits its ubiquitination, thereby reducing its degradation and maintaining protein stability. Under these conditions, propionate levels in the spinal microenvironment are decreased, thereby promoting a shift of microglia toward a pro-inflammatory state. AT-RvD1 treatment significantly suppresses Angptl4 expression, thereby weakening its stabilizing effect on Suclg1 and leading to downregulation of Suclg1, accompanied by an increase in propionate levels in the spinal microenvironment. These changes further promote a shift in microglial polarization from a pro-inflammatory toward an anti-inflammatory and pro-repair state, ultimately attenuating spinal inflammation and relieving pain hypersensitivity. In addition, the MG1-modified liposomal delivery system (AT-RvD1@Lipo-MG1) enables targeted delivery of AT-RvD1 to spinal microglia, thereby enhancing its anti-inflammatory and analgesic effects. Created in https://BioRender.com.

## 2 Experimental methods

### 2.1 Animals

Adult C57BL/6 J mice, male, aged 6-8 weeks, were sourced from Jinan Pengyue Laboratory Animal Breeding Co., Ltd. of China. A 12-h light/dark cycle was maintained for the mice, which were also provided with food and water ad libitum. Temperature and humidity were regulated at 23 ± 2 °C and 50-60%, respectively. These mice underwent randomization into distinct treatment groups.

### 2.2 Establishment of the nerve injury (SNI) model and drug administration

After anesthetizing the mice, a lateral thigh incision was performed, and the left sciatic nerve together with its three terminal branches was dissected free. The distal ends of both the common peroneal nerve and the tibial nerve were tied off using 5-0 sutures, and the distal ends were severed. The gastrocnemius nerve was not touched or pulled. In the sham group, the three terminal branches of the sciatic nerve were exposed without nerve injury [37]. To investigate the therapeutic effect of AT-RvD1 in the SNI model, intrathecal injections were administered at 1, 3, 5 and 7 days post-surgery, with a dose of 10 ng per mouse each time. For the propionate treatment group, mice received 200 mmol/L sodium propionate in the drinking water for two weeks, beginning on postoperative day 1.

### 2.3 Behavioral testing

#### 2.3.1 Vonfrey test

The mice were placed in a box with a high-pitched metal net. They were given 30 min of adaptation before the examination. A range of von Frey filaments with increasing hardness (0.02 - 2.56 g) were used to stimulate the mice's foot sole, perpendicular to the sole surface (each hair for 2 - 3 s). The 50% withdrawal response threshold was determined using the Dixon method. The tests were conducted before surgery (day 0) and on postoperative days 1, 3, 5, 7, and 14.

#### 2.3.2 Acetone experiment

Acetone was dropped on the toe surface of the hind paws. Each mouse underwent 5 experiments, with an interval of 5 min each. The degree and frequency of the mouse's paw withdrawal were scored: 0 point (no reaction: after the acetone drop, there was no obvious movement of the animal's paw); 1 points (mild/short reaction: the foot quickly and slightly lifted or trembled for a moment, then returned to the grid immediately, without paw licking behavior); 2 points (moderate reaction: the foot lifted, contracted, or trembled several times for a duration of 1 - 5 s, possibly accompanied by a brief paw licking); 3 points (strong/continuous reaction: prolonged lifting of the paw, paw licking, claw biting, or intense trembling, duration > 5 s, possibly accompanied by a scream or turning towards the stimulus area).

### 2.4 Determination of propionate by GC-MS

Homogenization of 0.05 g samples in 5% phosphoric acid (2 ml) was performed, and then extracted with 2 mL diethyl ether. Subsequently, the mixture was vortexed for 10 min, and followed by centrifugation under ice-bath conditions at 4000 rpm for 20 min. Collection of the organic phase was carried out, followed by a single additional extraction. The volume of the combined extracts was brought up to 4 mL for analysis. GC-MS analysis was performed using a TG-WAX column (30 m × 0.25 mm × 0.25 μm). The oven temperature was programmed to start at 100 °C for 5 min, increase to 160 °C at 5 °C/min, then rise to 240 °C at 30 °C/min and be held for 20 min. A flow rate of 1 mL/min and a split ratio of 20:1 were used for helium, which served as the carrier gas. The injector temperature was maintained at 240 °C. Using selected ion monitoring (SIM) under electron ionization (EI) at 70 eV, we conducted mass spectrometry. The ion source temperature was maintained at 200 °C, and the transfer line at 250 °C. To quantify propionate, the ions *m*/*z* 60 and 73 were used. Data analysis was conducted using Chromeleon software (Thermo Scientific, USA), and absolute quantification of propionate was performed based on standard calibration curves.

### 2.5 Cell culture

This study utilized the mouse microglial cell line (BV2). Cell culture was conducted in DMEM medium (PM150210, Procell Life Science & Technology, China), supplemented with 10% fetal bovine serum (164210, Procell Life Science & Technology, China) and 1% penicillin-streptomycin solution (PB180120, Procell Life Science & Technology, China). The cells were cultured in a humidified incubator at 37 °C and 5% CO2. Conventional subculture was performed when the cell confluence reached 80-90%.

### 2.6 CCK-8 assay

After seeding BV2 cells in a 96-well plate at 2000 cells/well with 100 μL of complete medium, the cells were treated with different concentrations of AT-RvD1 for 12 h, followed by the addition of 10 μL of CCK8 reagent (SC119-02, Sevenbio, China) per well and a further 2-h culture at 37 °C. Absorbance at 450 nm was then measured with a microplate reader (Berthold Technologies, Germany).

### 2.7 Construction of gene overexpression plasmid and transfection of siRNA

The Angptl4 overexpression plasmid was constructed using the pcDNA3.1(+) vector provided by Genepharma. The recombinant plasmid was transfected into cells using liposome transfection reagents. The siRNA sequence is as follows:

**Table:** 

Oligos	Sense 5′-3′	Antisense 5′-3′
siAngptl4-1	CCUGGGACGAGAUGAACUUTT	AAGUUCAUCUCGUCCCAGGTT
siAngptl4-2	GGAACCGAGGAAGCCAAUUTT	AAUUGGCUUCCUCGGUUCCTT
siSuclg1-1	GUCCUUCAUUGCUGGUAUATT	UAUACCAGCAAUGAAGGACTT
siSuclg1-2	GUAUUCCGCAGCAGGAUAUTT	AUAUCCUGCUGCGGAAUACTT
siNC	CACUCAAGAUUGUCAGCAATT	UUGCUGACAAUCUUGAGUGAG

### 2.8 Flow cytometry

The polarization status of BV2 cells was analyzed by flow cytometry using the M1-associated marker CD86 and the M2-associated marker CD206. The analysis was performed using a flow cytometer (powclin, China), and all flow cytometer data underwent analysis with FlowJo software (v10.8). The antibodies used are as follows: APC-CD206 (17-2061-80, Thermo Fisher Scientific, USA) and FITC-CD86 (11-0862-81, Thermo Fisher Scientific, USA).

### 2.9 Co-culture of BV2 with HT-22 cells

A 6-well plate with a 0.4-μm pore size Transwell chamber was used for the co-culture system of BV2 cells/HT-22 cells. In brief, BV2 cells subjected to different treatments were collected at a density of 1 × 10^5^ cells per well and loaded into the upper Transwell chamber, whereas for HT-22 cells, 5 × 10^5^ cells per well were seeded into a 6-well plate. The cells were then co-cultured at 37 °C for 24 h, after which qRT-PCR and Western blot were employed to analyze the HT-22 cells.

### 2.10 qRT-PCR

RNA was extracted using the Total RNA Extraction Kit (SM130, Sevenbio, China), and then complementary DNA (cDNA) was synthesized from the RNA using the Evo M-MLV reverse transcription premix Kit (AG11728, Accurate Biology, China). Gene expression was examined using Hieff qPCR SYBR Green Master Mix (No ROX) (11201ES03, Yeasen Biotechnology, China), Roche LightCycler® 96. The fold changes in gene expression were determined via the 2^−ΔΔCT^ method. Supplementary Table S1 contains all the primer sequences required for the qRT-PCR procedure.

### 2.11 Western blot

Following lysis of BV2 cells or spinal cord tissues in RIPA buffer (32010A, beibokit, China) with protease inhibitors, the protein was quantified by BCA kit (PC0020, Solarbio, China). After SDS-PAGE separation, the proteins were electrotransferred onto PVDF membranes (0.45 μm, Millipore, Germany) and blocked with 5% non-fat milk for 2 h, and incubated overnight at 4 °C with primary antibodies. After three washes with TBST, membranes were incubated with secondary antibodies at room temperature for 1.5 h [38]. The following primary antibodies were used for Western Blot: rabbit anti-iNOS (22226-1-AP, Proteintech, China), rabbit anti-ANGPTL4 (18374-1-AP, Proteintech, China), rabbit anti-SUCLG1 (86072-1-RR, Proteintech, China), rabbit anti-IL-1β (26048-1-AP, Proteintech, China), rabbit anti-ubiquitin (10201-2-AP, Proteintech, China) and mouse anti-IL-6 (26404-1-AP, Proteintech, China). The secondary antibodies were anti-rabbit HRP-conjugated (RGAM001, Proteintech, China) or anti-mouse HRP-conjugated recombinant secondary antibodies (RGAR001, Proteintech, China). The immunoblot signals were detected using a UVITEC-Alliance. GAPDH (60004-1-Ig, Proteintech, China) was used as an internal reference protein. Quantification was carried out with ImageJ software (National Institutes of Health, USA).

### 2.12 Immunofluorescence staining assay

The paraffin-embedded sections were first deparaffinized, rehydrated, washed, permeabilized, and blocked before staining. The primary antibodies used included anti-Iba1 (microglia marker), anti-CD86 (YM8023, Immunoway, USA), anti-CD206 (83485-1-RR, Proteintech, China), anti-IL-6, anti-IL-1β, and anti-ANGPTL4 antibodies. Spinal cord sections, after overnight incubation with primary antibodies at 4 °C, were then washed with PBS. Subsequently, the sections were incubated with secondary antibodies that were fluorescently labeled (RGAR004/RGAM002, diluted 1:500, Proteintech, China) for 2 h (RGAR004, Proteintech, China) at ambient temperature. DAPI was applied to counterstain the nuclei for 10 min following an additional wash [39].

At room temperature, after being fixed with 4% paraformaldehyde for 10 min and washed three times with PBS, the cells were permeabilized with 0.5% Triton X-100 for 10 min. To minimize non-specific binding, a blocking step was applied. The samples were incubated with 5% bovine serum albumin (BSA) at 37 °C for 1 h. Subsequently, in a 4 °C wet box, the corresponding primary antibodies were incubated overnight. On the following day, after washing three times with PBS, fluorescently labeled secondary antibodies (RGAR004/RGAM002, diluted 1:500, Proteintech, China) were added and incubated in the dark for 1 h. After washing with PBS, the nuclei were stained with DAPI solution for 10 min. Finally, the slides were mounted with anti-fade mounting medium. Images were collected using a laser confocal microscope, and the excitation light, exposure time, and gain conditions were kept the same for all groups. ImageJ software served as the tool for image analysis, and the fluorescence intensity was quantified using the average gray value.

### 2.13 ELISA assay

The levels of IL-6 and IL-1β expression in AT-RvD1 and different treated microglial cells or spinal cord tissue were detected using the Mouse IL-6 and IL-1β ELISA Kit (EM0004/EM0029, youkelife, China). Measurement of the absorbance (OD) values at 450 nm was performed according to the manufacturer's instructions.

### 2.14 Co-immunoprecipitation (Co-IP)

For each IP reaction, 400 μL of total protein lysis buffer were used. After co-incubation of Protein A/G magnetic beads (HY-K0202, MedChemExpress, USA) with the specific antibody or isotype control IgG (5 μg/mL, 66360-1-Ig, Proteintech, China), the whole cell lysate was centrifuged at 14,000 rpm for 10 min at 4 °C to remove cell debris [40]. The resulting preparation was combined with the supernatant and incubated at 4°C for 2h. Anti-ANGPTL4 and anti-SUCLG1 were used to pull down the complex. The complex was washed with phosphate-buffered saline (PBST) and analyzed by Western blot. The gel was subsequently stained with the Fast Silver Stain Kit (P0017S, Beyotime, China) as per the manufacturer's instructions.

### 2.15 Quantitative proteomics analysis

The quantitative proteomics analysis was conducted using the data-independent acquisition (DIA) based quantitative proteomics technique. The cell specimens were lysed using urea lysis buffer, subjected to ultrasonic treatment, and then centrifuged at 4 °C. The BCA method was used to determine the protein concentration in the collected supernatant. Identical quantities of protein were reduced with DTT and alkylated with iodoacetamide, and then digested overnight with trypsin at 37 °C. The obtained peptide segments were desalted using a C18 column for LC-MS/MS analysis. The peptide separation was performed using the Vanquish Neo nano-scale liquid chromatography system, and the mass spectrometry detection was carried out in the Orbitrap Astral mass spectrometer in positive ion mode and DIA mode. Analysis of the mass spectrometry data was performed by means of DIA-NN (v1.8.1) software in the non-spectrum library mode, and the search library was conducted using the UniProt database. Application of a 1% false discovery rate (FDR) to filter the precursor ions and protein levels was followed by protein quantification completion via the MaxLFQ algorithm. The criteria for differentially expressed proteins were defined as those meeting a fold change of ≥ 1.5 or ≤ 0.67 and a P-value < 0.05. The differentially expressed proteins were functionally annotated and analyzed for pathway enrichment based on the GO and KEGG databases.

### 2.16 Immunoprecipitation combined with mass spectrometry (IP-MS)

After the cell lysis solution was pre-filtered, specific antibodies were added to Protein A/G magnetic beads and incubated at 4 °C for immunoprecipitation. A thorough wash was applied to the magnetic beads, and the immune complexes were eluted. The eluted samples were treated with urea lysis buffer for protein reduction (DTT) and alkylation (IAA), and then 1:50 enzyme-to-substrate ratio trypsin was added and digested at 37 °C for approximately 20 h. The digested peptide segments were desalted, freeze-dried, and re-solubilized in 0.1% formic acid solution for subsequent mass spectrometry analysis. The peptide segments were separated using a nanoElute nanoscale liquid chromatography system and detected on a timsTOF Pro high-resolution mass spectrometer (Bruker). Positive ion mode was used for mass spectrometry analysis, and parallel accumulation serial fragmentation (PASEF) mode was employed for data acquisition. The raw mass spectrometry data were retrieved and protein identified using the MaxQuant software (v1.6.14).

### 2.17 Preparation, characterization, and targeted delivery of AT-RvD1-loaded MG1-Modified liposomes (A@Lipo-MG1)

DSPE-PEG_2000_-NHS was conjugated with CHHSSSARC (MG1 peptide, Lot: RM0252764) by dissolving DSPE-PEG_2000_-NHS (30 mg) and MG1 peptide (10 mg) in 2 mL of DMF and stirring the mixture for 24 h at room temperature. The reaction mixture was dialyzed against deionized water for 48 h and lyophilized under vacuum to obtain DSPE-PEG_2000_-MG1. For liposome preparation, soybean phosphatidylcholine (40 mg), cholesterol (10 mg), DSPE-PEG_2000_-MG1 (10 mg), and AT-RvD1 (1 μg) were dissolved in 2 mL of anhydrous ethanol. A uniform lipid thin film was formed by rotary evaporation of the solvent at 60 °C. The film was then hydrated with 1 mL of deionized water and extruded through a 50 nm polycarbonate membrane using a liposome extruder, yielding A@Lipo-MG1 nanoparticles.

Characterization of the liposomes’ physicochemical properties was performed by dynamic light scattering (DLS) and zeta potential measurements. Liposome suspensions were diluted 10-fold with PBS and analyzed at 25 °C. Triplicate measurements were performed for hydrated particle size, polydispersity index (PDI), and zeta potentia, and mean values were reported. Liposome morphology was examined using TEM after negative staining with 2% phosphotungstic acid. In addition, after dissolving MG1 peptide, DSPE-PEG_2000_-NHS, and DSPE-PEG_2000_-MG1 in deuterated chloroform (CDCl_3_), thirty-two ^1^H NMR scans over a chemical shift range of 0-10 ppm were recorded to confirm successful conjugation.

For in vitro cellular uptake, after seeding in 6-well plates, BV2 microglial cells were cultured to approximately 70% confluence. The above cells were exposed to Rhodamine B (RhB)@Lipo or RhB@Lipo-MG1 (final RhB concentration: 10 μg/mL), while the control group received free RhB solution. Following 1 h and 6 h incubations at 37 °C, cells were washed in triplicate with PBS, with intracellular fluorescence being observed using a fluorescence microscope. In parallel, flow cytometry was performed on collected cells to quantify mean fluorescence intensity as a readout of cellular uptake efficiency.

For in vivo targeted delivery, SNI mice were randomly assigned to three groups: free IR780, IR780@Lipo, and IR780@Lipo-MG1. The formulations were administered via intravenous injection at a dose equivalent to 1 mg/kg IR780. In vivo fluorescence distribution was monitored at 6 h and 24 h post-injection using a small animal live imaging system. At 24 h after injection, mice were euthanized, and the spinal cords were collected for ex vivo fluorescence imaging. For fluorescence co-localization analysis, RhB-labeled formulations were administered intravenously to SNI mice. The L4-L6 spinal cord segments were collected, prepared as frozen sections, immunostained for Iba1, and examined using confocal laser scanning microscopy to assess the co-localization of RhB-labeled liposomes with microglia.

### 2.18 Determination of AT-RvD1 by LC-MS/MS

AT-RvD1 concentrations in tissue and liquid samples were determined by UPLC-MS/MS using an external standard method. Samples were extracted with LC-MS-grade methanol, centrifuged at 13,000 rpm for 20 min at 4 °C, and filtered through a 0.22 μm membrane before analysis. Chromatographic separation was performed on a C18 column using 0.1% formic acid in water and acetonitrile as the mobile phases, and AT-RvD1 was quantified in multiple reaction monitoring (MRM) mode.

### 2.19 Statistical analysis

All statistical analyses were performed using GraphPad Prism 9.0. Data are presented as mean ± standard deviation (SD). For two-group comparisons, Student's t-test was used. For comparisons among multiple groups, one-way ANOVA followed by Tukey's multiple-comparisons test was performed. For behavioral data involving repeated measurements over time, two-way repeated-measures ANOVA followed by Bonferroni's multiple comparisons test was used to account for within-subject correlations. A P value < 0.05 was considered statistically significant.

## 3 Results

### 3.1 AT-RvD1 alleviates hypersensitivity and spinal neuroinflammation in spared nerve injury (SNI) mice

To evaluate the effect of AT-RvD1 on alleviating hypersensitivity in SNI mice, we measured the mechanical pain threshold (PWT) using the von Frey test and assessed cold sensitivity through the acetone stimulation assay on days 0, 1, 3, 5, 7, and 14 after SNI treatment (Fig. 2A). The results showed that, relative to the sham group, the SNI group exhibited a significant decrease in PWT and a marked increase in acetone test scores from day 1 post-surgery, indicating successful establishment of the NP model. Additionally, AT-RvD1 administration significantly increased PWT and decreased acetone test scores on days 1, 3, 5, 7, and 14 post-surgeries, demonstrating that AT-RvD1 effectively alleviates SNI-induced hypersensitivity (Fig. 2B). On day 14 post-surgery, L4-L6 spinal cord segments were collected for molecular and histological analyses. From the western blot analysis results, the protein levels of pro-inflammatory mediators IL-6 and IL-1β, as well as the pro-inflammatory microglial marker inducible nitric oxide synthase (iNOS), were significantly elevated in the SNI group relative to the sham group, whereas AT-RvD1 treatment markedly reversed these changes (Fig. 2C, Fig. S1A). Immunofluorescence staining of spinal cord sections further demonstrated a marked increase in the microglial marker Iba1, as well as elevated IL-6 and IL-1β levels in the SNI group, all of which were markedly reduced following AT-RvD1 administration(Fig. 2D).

**Fig. 2:**
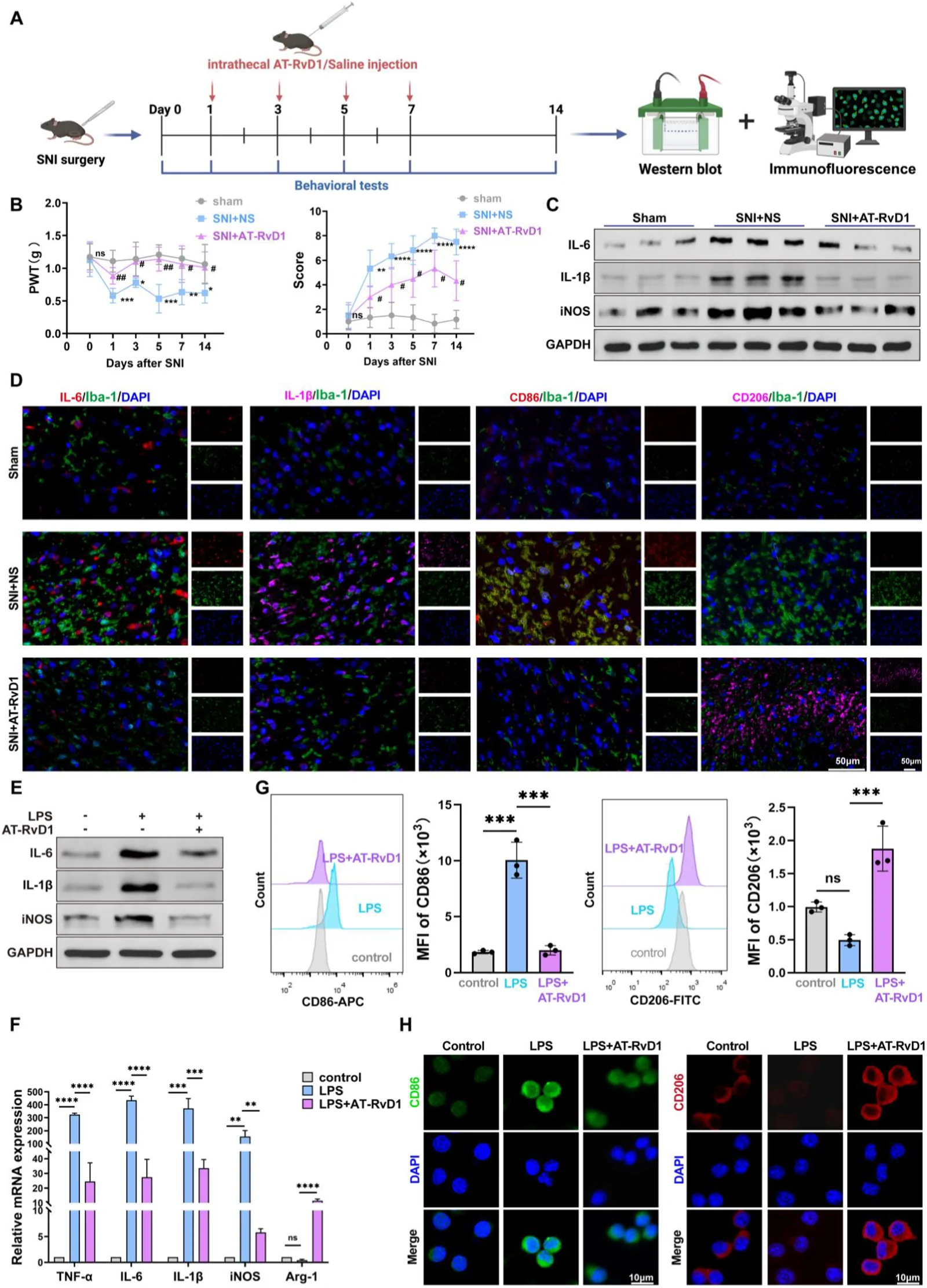
**Effects of AT-RvD1 on SNI mice and LPS-induced inflammatory responses in BV2 cells. (A)** Schematic diagram of the experimental design for intrathecal administration of AT-RvD1 in SNI mice. Behavioral tests are performed at the indicated time points, and mice are sacrificed on day 14 after surgery for tissue collection.Created in https://BioRender.com. **(B)** PWT (g) and acetone tests were used to assess the efficacy of AT-RvD1 on mechanical allodynia and cold hypersensitivity in SNI mice (n = 6).**P < 0.01, ***P < 0.001, ****P < 0.0001 vs. Sham group; #P < 0.05, ##P < 0.01. **(C)** The protein expressions of IL-6, IL-1β, and iNOS in spinal cord tissues from different groups by western blot analysis. **(D)** Representative immunofluorescence images showing IL-6 (red), IL-1β (purple), Iba1 (green), CD86 (red), and CD206 (purple) in the spinal dorsal horn. Scale bar = 50 μm. **(E)** The protein expressions of IL-6, IL-1β, and iNOS in BV2 cells by western blot analysis. **(F)** Total RNA is extracted from BV2 cells, and the mRNA levels of TNF-α, IL-6, IL-1β, iNOS, and Arg-1 are determined by qRT-PCR. **(G)** The mean fluorescence intensity (MFI) of CD86 and CD206 in BV2 microglial cells was measured by flow cytometry and analyzed via FlowJo software (v10.0). **(H)** Representative immunofluorescence images showing CD86 (green) and CD206 (red) expression in BV2 cells. Scale bar = 10 μm. Data are presented as mean ± SD (n = 3 per group). *P < 0.05, **P < 0.01, ***P < 0.001, and ****P < 0.0001 indicate statistically significant differences between groups. (For interpretation of the references to colour in this figure legend, the reader is referred to the Web version of this article.)

Under physiological conditions, the dynamic balance of microglial polarization maintains CNS homeostasis and supports repair processes, whereas dysregulation of this balance drives neuroinflammation and NP progression. We therefore performed immunofluorescence co-staining for the M1-associated marker CD86 and M2-associated marker CD206 in spinal cord sections to assess microglial polarization states. The results showed that CD86 expression was significantly increased in the SNI group compared with the sham group, whereas AT-RvD1 treatment markedly decreased CD86 expression while increasing CD206 expression relative to the SNI group. (Fig. 2D, Fig. S1B). These findings suggest that AT-RvD1 facilitates microglial polarization from a pro-inflammatory toward an anti-inflammatory and pro-repair state, which consequently alleviates nociceptive hypersensitivity in NP mice.

### 3.2 AT-RvD1 attenuates pro-inflammatory responses and regulates microglial inflammatory responses in vitro

To further evaluate the regulatory effects of AT-RvD1 on microglia, we cultured BV2 microglial cells in vitro, and performed CCK-8 assays to assess the cytotoxicity of AT-RvD1. The results indicated that AT-RvD1 had no significant effect on cell survival at concentrations less than 200 nM (Fig. S1C). To detect the anti-inflammatory efficacy of AT-RvD1, we triggered microglial activation with LPS, followed by treatment with three concentrations of AT-RvD1 (10 nM, 30 nM, and 50 nM). The results showed that AT-RvD1 suppressed LPS-induced inflammatory responses in a concentration-dependent manner, with the strongest efficacy observed at 50 nM (Fig. S1D and E), which was selected for all subsequent in vitro experiments. Western blot analysis showed that IL-6, IL-1β, and iNOS protein levels were substantially increased in the LPS-stimulated group, whereas AT-RvD1 treatment markedly suppressed these LPS-induced upregulations (Fig. 2E, Fig. S1F). Consistently, qRT-PCR analysis showed that LPS stimulation considerably increased the mRNA levels of pro-inflammatory mediators, including TNF-α, IL-6, IL-1β, and iNOS, whereas AT-RvD1 treatment markedly reversed these effects. Meanwhile, the mRNA expression of the M2-associated marker arginase-1 (Arg-1) was substantially increased in the AT-RvD1-treated group relative to the LPS group (Fig. 2F). Enzyme-linked immunosorbent assay (ELISA) results aligned with the mRNA and protein findings (Fig. S1G), confirming that AT-RvD1 effectively suppresses LPS-induced pro-inflammatory responses in microglia. Afterward flow cytometry analysis revealed that LPS treatment substantially increased CD86 expression, indicating enhanced pro-inflammatory polarization in BV2 cells. In contrast, AT-RvD1 administration markedly downregulated CD86 expression and upregulated CD206 expression (Fig. 2G), indicating that AT-RvD1 promotes a microglial phenotypic shift from a pro-inflammatory toward an anti-inflammatory and pro-repair state. Immunofluorescence staining of CD86 and CD206 in BV2 cells further validated these regulatory effects of AT-RvD1 on microglial polarization (Fig. 2H, Fig. S1H).

### 3.3 AT-RvD1 promotes anti-inflammatory microglial polarization and exerts analgesic effects via inhibiting Angptl4 expression

To identify candidate molecular targets potentially involved in the regulatory effects of AT-RvD1 on microglial activation states, we downloaded the GSE634428 dataset from the Gene Expression Omnibus (GEO) database, which was generated from a spinal nerve ligation (SNL) model of neuropathic pain and corresponding sham controls. Differential expression analysis between the sham and SNL groups identified several differentially expressed genes (DEGs), including Angptl4, Xbp1, Jun, Hsf4, and Trim2 (Fig. 3A). To validate the selected differentially expressed genes, qRT-PCR was subsequently carried out in BV2 cells. (Fig. S2A). According to the results, LPS stimulation significantly upregulated Angptl4 mRNA expression, whereas AT-RvD1 treatment markedly suppressed the LPS-induced upregulation of Angptl4 (Fig. 3B). Furthermore, Western blotting and immunofluorescence analyses revealed that Angptl4 protein expression was markedly enhanced in the LPS-stimulated group, whereas its expression was significantly reversed by AT-RvD1 treatment (Fig. 3C and E, Figs. S2 B and C). Consistently, in vivo results showed that AT-RvD1 significantly reduced Angptl4 expression in the spinal cord of SNI mice (Fig. 3D and F, Figs. S2D and E), indicating that AT-RvD1 effectively suppresses the upregulation of Angptl4 during neuroinflammation.

**Fig. 3:**
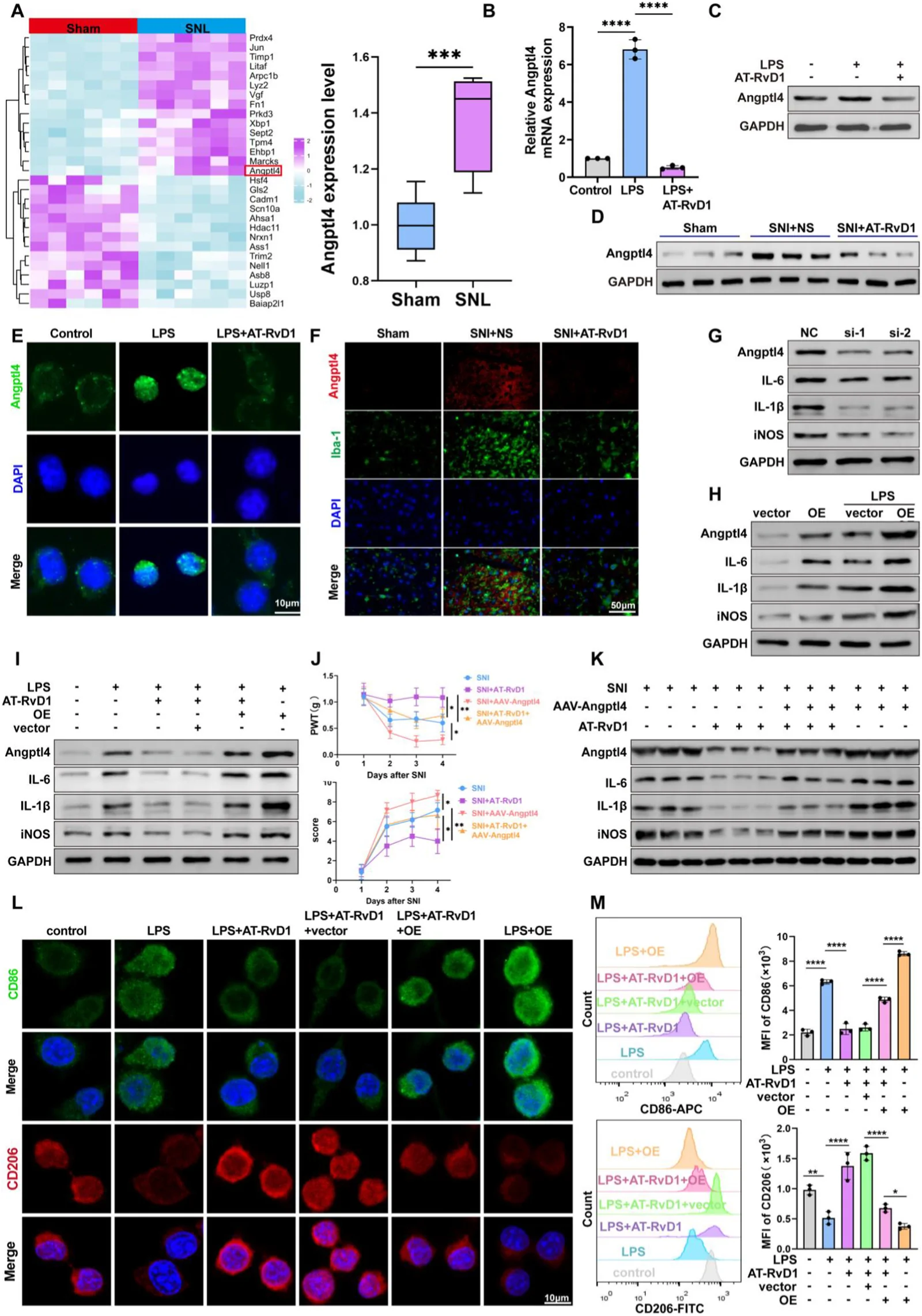
**AT-RvD1 alleviates pain hypersensitivity by inhibiting Angptl4 expression and regulating microglial polarization. (A)** Heatmap of differentially expressed genes between Sham and spinal nerve ligation (SNL) mice, and Angptl4 differential expression analysis. Upregulated genes are represented in purple and downregulated ones in blue (P < 0.05). **(B)** qRT-PCR analysis of Angptl4 mRNA expression in BV2 cells from control, LPS, and LPS + AT-RvD1 groups. **(C-D)** Western blot assay of Angptl4 protein levels in BV2 cells (C) or mouse spinal cord tissues (D) under different treatments. **(E)** Representative immunofluorescence staining of Angptl4 (green) in BV2 microglial cells. Scale bar = 10 μm. **(F)** Representative immunofluorescence micrographs showing Angptl4 expression (red) in the spinal dorsal horn of mice. Scale bar = 50 μm. **(G)** Western blot assay of Angptl4, IL-6, IL-1β, and iNOS protein levels in BV2 cells transfected with si-Angptl4. **(H)** Western blot analysis of Angptl4, IL-6, IL-1β, and iNOS protein levels in BV2 cells transfected with vector or Angptl4 overexpression plasmid (OE), with or without LPS stimulation. **(I)** Representative Western blot images showing Angptl4, IL-6, IL-1β, and iNOS protein expression in different treatment groups. **(J)** PWT(g) and acetone test scores in SNI mice treated with AT-RvD1, AAV-Angptl4, or their combination (n = 6). **(K)** Western blot assay of Angptl4, IL-6, IL-1β, and iNOS protein levels in spinal cord tissues from different treatment groups. **(L)** Immunofluorescence images showing CD86 (green) and CD206 (red) expression in BV2 cells under different treatments. Scale bar = 10 μm. **(M)** The proportions of CD86-positive and CD206-positive BV2 cells were analyzed by flow cytometry, and processed via FlowJo software (v10.0). Data are presented as mean ± SD (n = 3 per group). *P < 0.05,**P < 0.01,***P < 0.001,****P < 0.0001 represent statistically significant differences between groups. (For interpretation of the references to colour in this figure legend, the reader is referred to the Web version of this article.)

To clarify the role of Angptl4 in microglial inflammation and polarization, we knocked down Angptl4 expression in BV2 cells using small interfering RNA (siRNA). Both siAngptl4-1 and siAngptl4-2 effectively reduced Angptl4 mRNA and protein levels (Fig. 3G, Fig. S2F), confirming high knockdown efficiency. Functional assays showed that Angptl4 knockdown substantially suppressed the expression of IL-6, IL-1β, and iNOS (Fig. 3G, Fig. S2G), suggesting a key pro-inflammatory role of Angptl4 in microglial activation. Next, we constructed an Angptl4 overexpression plasmid, which markedly increased Angptl4 mRNA and protein expression levels (Fig. 3H, Fig. S2H). Overexpression of Angptl4 enhanced pro-inflammatory cytokine levels, such as IL-6 and IL-1β (Fig. 3H, Fig. S2I), and attenuated the anti-inflammatory efficacy of AT-RvD1 in vitro (Fig. 3I, Fig. S2J), suggesting that Angptl4 interferes with AT-RvD1-mediated suppression of inflammation. To validate these findings in vivo, Angptl4 was delivered to SNI mice via adeno-associated virus (AAV). The AAV-Angptl4 group exhibited higher levels of pro-inflammatory factors than the AT-RvD1 treated group, along with significantly reduced mechanical and cold pain thresholds (Fig. 3J and K, Fig. S2K), indicating that Angptl4 overexpression can counteract the analgesic effects of AT-RvD1. Moreover, flow cytometry and immunofluorescence analyses of BV2 polarization revealed that Angptl4 overexpression significantly promoted pro-inflammatory polarization while suppressing anti-inflammatory and pro-repair polarization. Compared with the AT-RvD1-treated group, it inhibited AT-RvD1-induced shift from a pro-inflammatory toward an anti-inflammatory and pro-repair state. (Fig. 3L and M, Fig. S2L). In summary, Angptl4 serves as a key target mediating AT-RvD1-regulated microglial polarization. By suppressing Angptl4 expression, AT-RvD1 facilitates a microglial phenotypic shift from a pro-inflammatory toward an anti-inflammatory and pro-repair state, which in turn yields anti-inflammatory and analgesic benefits.

### 3.4 AT-RvD1 downregulates Suclg1 expression via inhibiting Angptl4

To further delineate the downstream molecular mechanisms of the AT-RvD1/ANGPTL4 axis in regulating microglial polarization, we performed proteomic profiling of BV2 cells under different treatment conditions, followed by Gene Set Enrichment Analysis (GSEA). According to the results, AT-RvD1 treatment substantially modulated the propionate metabolism pathway (Fig. 4A–C). Furthermore, we measured propionate levels in the spinal cord by the way of Gas Chromatography-Mass Spectrometry (GC-MS), and found that AT-RvD1 administration significantly increased spinal propionate concentrations (Fig. 4D).

**Fig. 4:**
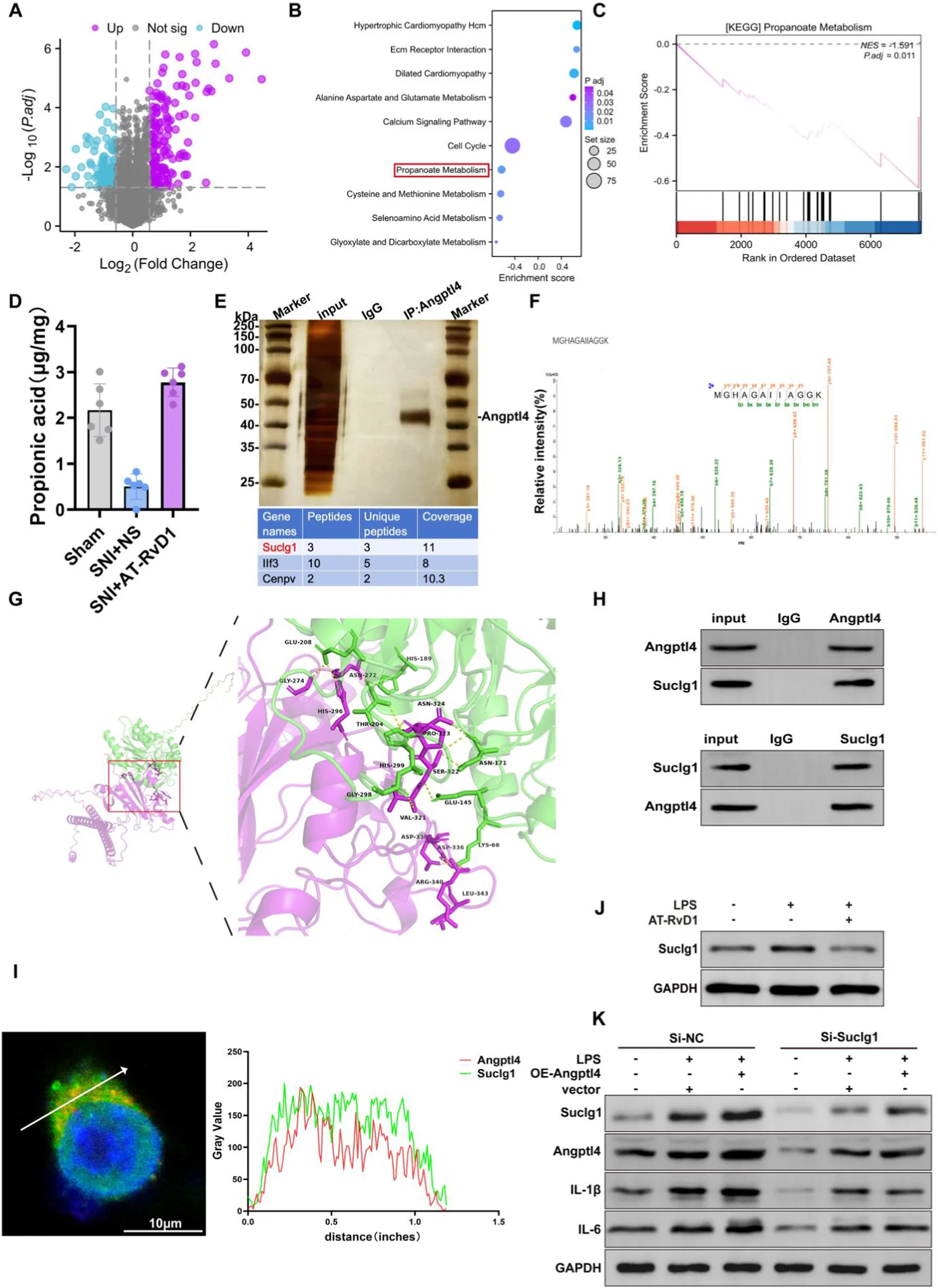
**AT-RvD1 suppresses Suclg1 expression via Angptl4. (A)** Volcano plot showing differentially expressed genes in BV2 cells treated with AT-RvD1 following LPS stimulation. Significantly upregulated and downregulated genes are indicated in purple and blue, respectively. **(B)** GSEA-KEGG pathway enrichment analysis comparing AT-RvD1 and LPS groups. **(C)** GSEA enrichment plot of the propionate metabolism pathway (P < 0.05). **(D)** Propionate levels in the spinal cord under different treatment conditions were measured using GC-MS (n = 6). **(E)** IP with IgG or an anti-Angptl4 antibody, followed by silver staining, was performed in BV2 cells. Potential interacting partners of Angptl4 were then identified by IP-MS, with representative peptides and sequence coverage of Suclg1 and other candidate proteins shown. **(F)** Mass spectrometry analysis identifying a representative peptide of Suclg1.**(G)**Predicted molecular docking model illustrating hydrogen-bond interactions between Angptl4 and Suclg1. **(H)** BV2 cell lysates were immunoprecipitated with Angptl4 or Suclg1 antibodies, followed by Western blot analysis. **(I)** Confocal immunofluorescence images showing co-localization of Angptl4 (red) and Suclg1 (green) in BV2 cells, and the intensity of fluorescence was determined. Scale bar = 10 μm. **(J)** Suclg1 protein expression in BV2 cells by western blot analysis. **(K)** Western blot analysis of Suclg1, Angptl4, IL-6, and IL-1β protein levels in BV2 cells under different treatments. Data are presented as mean ± SD (n = 3 per group). *P < 0.05, **P < 0.01, ***P < 0.001, ****P < 0.0001 represent statistically significant differences between groups. (For interpretation of the references to colour in this figure legend, the reader is referred to the Web version of this article.)

To identify potential interacting proteins of Angptl4, we performed immunoprecipitation followed by Western blot (IP-WB) and silver staining analysis (Fig. 4E). Silver staining of the Angptl4 IP samples revealed a prominent protein band, which was absent in the IgG control, indicating successful enrichment of Angptl4-associated proteins. Subsequently, immunoprecipitation coupled with mass spectrometry (IP-MS) analysis suggested that Suclg1 may serve as a binding partner of Angptl4 (Fig. 4E and F). Suclg1 is a key enzyme in propionate metabolism, and previous studies have demonstrated that Suclg1 inhibition significantly reduces inflammatory responses in macrophages, suggesting its important role in metabolic regulation and immune responses [41]. Molecular docking analysis revealed specific hydrogen bond interactions between key amino acid residues of Angptl4 and Suclg1 (Fig. 4G). To further validate the interaction between Angptl4 and Suclg1, co-immunoprecipitation (Co-IP) combined with Western blot analysis was performed, which confirmed their direct interaction (Fig. 4H). In addition, immunofluorescence co-localization assays demonstrated a clear co-localization of Angptl4 and Suclg1 within cells (Fig. 4I).

To evaluate the regulatory effect of AT-RvD1 on Suclg1, we performed Western blot analysis. The results indicated that Suclg1 expression was substantially upregulated in LPS-treated cells in contrast to the control group, whereas AT-RvD1 treatment markedly downregulated its expression (Fig. 4J, Fig. S3A). Subsequently, we knocked down Suclg1 in BV2 cells using siRNA and confirmed the knockdown efficiency (Fig. S3B). Functional validation experiments were then performed by overexpressing Angptl4 in Suclg1-deficient cells. Knockdown of Suclg1 substantially decreased the expression of IL-6, IL-1β, and iNOS. In the si-NC control group, Angptl4 overexpression upregulated Suclg1 levels and markedly increased IL-6, IL-1β, and iNOS levels; however, in the si-Suclg1 group, Angptl4 overexpression did not result in significant increases in these pro-inflammatory factors (Fig. 4K, Fig. S3C). Accordingly, these findings indicate that AT-RvD1 regulates the expression of the downstream Angptl4-interacting protein Suclg1 by suppressing Angptl4, thereby preventing the production of pro-inflammatory factors in microglia and revealing a cascade regulatory relationship among AT-RvD1, Angptl4, and Suclg1.

### 3.5 Angptl4 regulates Suclg1 degradation via the ubiquitin-proteasome pathway

To explore how Angptl4 regulates Suclg1, we examined changes in Suclg1 protein levels following Angptl4 knockdown or overexpression. The results showed that Angptl4 silencing markedly reduced Suclg1 protein expression, whereas Angptl4 overexpression significantly increased Suclg1 protein levels (Fig. 5A and B, Figs. S4A and B). Notably, Suclg1 mRNA levels remained unchanged under either Angptl4 knockdown or overexpression (Fig. S4C and D), suggesting that Angptl4 primarily modulates Suclg1 protein stability through a post-transcriptional mechanism. To further determine the degradative pathway involved, we inhibited protein synthesis with cycloheximide (CHX) and treated BV2 cells with either the proteasome inhibitor MG132 or the lysosomal inhibitor chloroquine (CQ). MG132 treatment markedly prolonged the half-life of Suclg1, whereas CQ had no significant effect, indicating that Suclg1 degradation predominantly occurs through the ubiquitin-proteasome pathway (Fig. 5C and D).

**Fig. 5:**
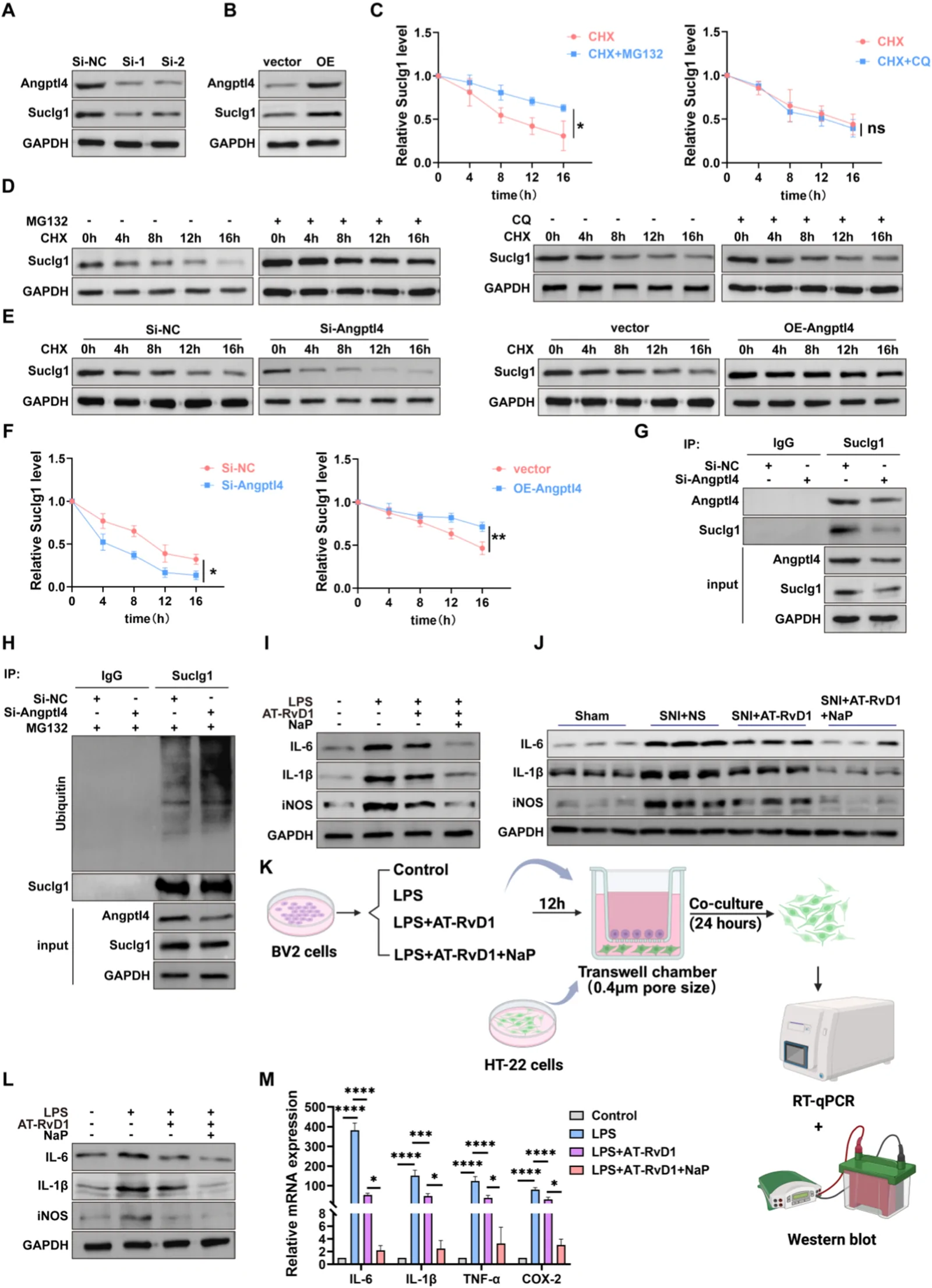
**Angptl4 regulates Suclg1 degradation via the ubiquitin-proteasome pathway. (A)** Suclg1 protein expression in Angptl4-knockdown BV2 cells by western blot analysis. **(B)** Suclg1 protein expression in Angptl4-overexpressing BV2 cells by western blot analysis. **(C-D)** BV2 cells were treated with CHX (20 μg/mL) alone or in combination with MG132 (10 μM) or CQ (50 μM) for the indicated times. Suclg1 protein levels were analyzed by Western blot, and band intensities were quantified. **(E)** Suclg1 protein levels in CHX-treated BV2 cells with Angptl4 knockdown or overexpression. **(F)** Quantification of Suclg1 band intensities in (E). **(G)** Denaturing IP and immunoblotting (IB) of Suclg1 in Angptl4-knockdown BV2 cells. **(H)** Ubiquitination assay: denaturing IP/IB of Suclg1 in MG132-treated, Angptl4-knockdown BV2 cells. **(I)** IL-6, IL-1β, and iNOS protein expression in BV2 cells under different treatments by western blot analysis. **(J)** IL-6, IL-1β, and iNOS protein expression in the spinal cord of SNI mice under different treatments by western blot analysis. **(K)** Schematic diagram of BV2 cell and HT-22 neuron co-culture experiments under different treatment conditions. Created in https://BioRender.com. **(L)** IL-6, IL-1β, and iNOS protein expression in BV2 cells from the co-culture experiments by western blot analysis. **(M)** qRT-PCR analysis of IL-6, IL-1β, TNF-α, and COX-2 mRNA expression in BV2 cells under different treatments. Data are presented as mean ± SD (n = 3 per group). *P < 0.05, **P < 0.01, ***P < 0.001, ****P < 0.0001 represent statistically significant differences between groups.

With the goal of further understanding the molecular basis underlying Angptl4-dependent modulation of Suclg1 protein stability, we treated BV2 cells with CHX after Angptl4 knockdown or overexpression, and examined Suclg1 protein levels over time. Angptl4 silencing markedly shortened the half-life of Suclg1, whereas Angptl4 overexpression significantly prolonged SUCLG1 half-life (Fig. 5E and F), confirming that Angptl4 stabilizes the Suclg1 protein. Co-IP assays showed that Angptl4 knockdown significantly weakened the protein-protein interaction between Angptl4 and Suclg1 (Fig. 5G). Furthermore, Co-IP analysis revealed that Angptl4 silencing markedly increased the polyubiquitination level of Suclg1 (Fig. 5H), indicating that Angptl4 protects Suclg1 from ubiquitin-proteasome-mediated degradation.

To further validate the functional role of propionate in microglial inflammatory responses, we treated LPS-stimulated BV2 cells with AT-RvD1 alone or in combination with sodium propionate (NaP). Compared with LPS treatment alone, combined administration of NaP and AT-RvD1 markedly downregulated pro-inflammatory cytokine expression, with a stronger inhibitory effect than AT-RvD1 monotherapy (Fig. 5I, Fig. S4E). Consistently, in the SNI mice model, the addition of NaP to AT-RvD1 treatment further reduced spinal inflammatory protein expression compared with AT-RvD1 treatment alone (Fig. 5J, Fig. S4F), and behavioral assessments further demonstrated enhanced alleviation of mechanical and cold allodynia (Fig. S4G). We then established a BV2 microglia-HT22 neuron co-culture chamber to assess the influence of microglial modulation on neuronal inflammation. BV2 cells were seeded in the upper chamber of Transwell inserts following various treatments, while HT22 neurons were cultured in the lower chamber of 6-well plates (Fig. 5K). The results suggested that LPS-stimulated BV2 cells significantly upregulated the levels of pro-inflammatory cytokines in co-cultured HT22 neurons. In contrast, AT-RvD1-treated BV2 cells markedly reduced pro-inflammatory cytokine expression in the underlying HT22 neurons, and this inhibitory effect was further enhanced by combined treatment with NaP and AT-RvD1 (Fig. 5L and M, Fig. S4H). These results suggest that Angptl4 stabilizes Suclg1 by interacting with it and inhibiting its polyubiquitination, thereby preventing proteasomal degradation. AT-RvD1 suppresses Angptl4 expression, thereby promoting the ubiquitination and degradation of Suclg1 and subsequently increasing propionate levels in the spinal cord. Propionate and AT-RvD1 jointly suppress microglia-neuron inflammatory signaling to reduce neuroinflammation and pain hypersensitivity. This study identifies a novel ANGPTL4-SUCLG1-propionate axis in AT-RvD1-regulated microglial inflammatory responses, offering a target for NP therapy.

### 3.6 A@Lipo-MG1 shows favorable physicochemical properties and efficient in vitro targeting capability

Given the clinical limitations of intrathecal AT-RvD1 administration, we constructed an MG1 peptide-modified liposome encapsulating AT-RvD1 (A@Lipo-MG1) to enable targeted intravenous delivery of AT-RvD1 to spinal microglia (Fig. 6A). Results from Nuclear magnetic resonance (^1^H NMR) spectroscopy demonstrated that MG1 was successfully conjugated to DSPE-PEG_2000_-NHS. Specifically, comparison of the MG1 and DSPE-PEG_2000_-NHS, the ^1^H NMR spectra of DSPE-PEG_2000_-MG1 showed the characteristic peaks of both MG1 and DSPE-PEG_2000_-NHS, verifying the successful synthesis of DSPE-PEG_2000_-MG1 (Fig. 6B). To evaluate the physicochemical properties of the liposomes, we measured their particle size and zeta potential, and further characterized their morphology through transmission electron microscopy (TEM). The particle size of A@Lipo and A@Lipo-MG1 were 58.42 ± 6.61 nm and 70.95 ± 4.95 nm, respectively (Fig. 6C). In addition, the polydispersity index (PDI) of both liposomes was below 0.3, indicating a uniform particle size distribution. Zeta potential measurements showed that A@Lipo exhibited a surface charge of −38.53 mV, which remained largely unchanged after conjugation with the targeting peptide (−38.22 mV), indicating that peptide modification had minimal impact on the liposomal surface charge and that the formulation maintained good stability. TEM was used to observe the morphology of the liposomes.The results showed that both A@Lipo and A@Lipo-MG1 exhibited a regular spherical morphology with intact membrane structures (Fig. 6D). The AT-RvD1-loaded liposomes exhibited an encapsulation efficiency (EE%) of 59.67 ± 7.91% and a drug loading (DL%) of 0.001%. In vitro release analysis showed cumulative AT-RvD1 release of 17.70 ± 2.45% at 1 h, 27.02 ± 1.88% at 2 h, followed by a more pronounced increase to 54.75 ± 2.30% at 4 h. Thereafter, the release gradually slowed, reaching 58.02 ± 2.09% at 8 h and 58.82 ± 2.15% at 24 h (Fig. 6E). These findings indicate satisfactory drug encapsulation and a controlled-release profile of the liposomal formulation. Collectively, these physicochemical characterizations demonstrate that A@Lipo-MG1 liposomal nanoparticles were successfully prepared in this study. The liposomes displayed uniform particle size, regular morphology, good stability, satisfactory AT-RvD1 encapsulation, and a controlled-release profile, providing a solid basis for subsequent in vitro and in vivo experiments.

**Fig. 6:**
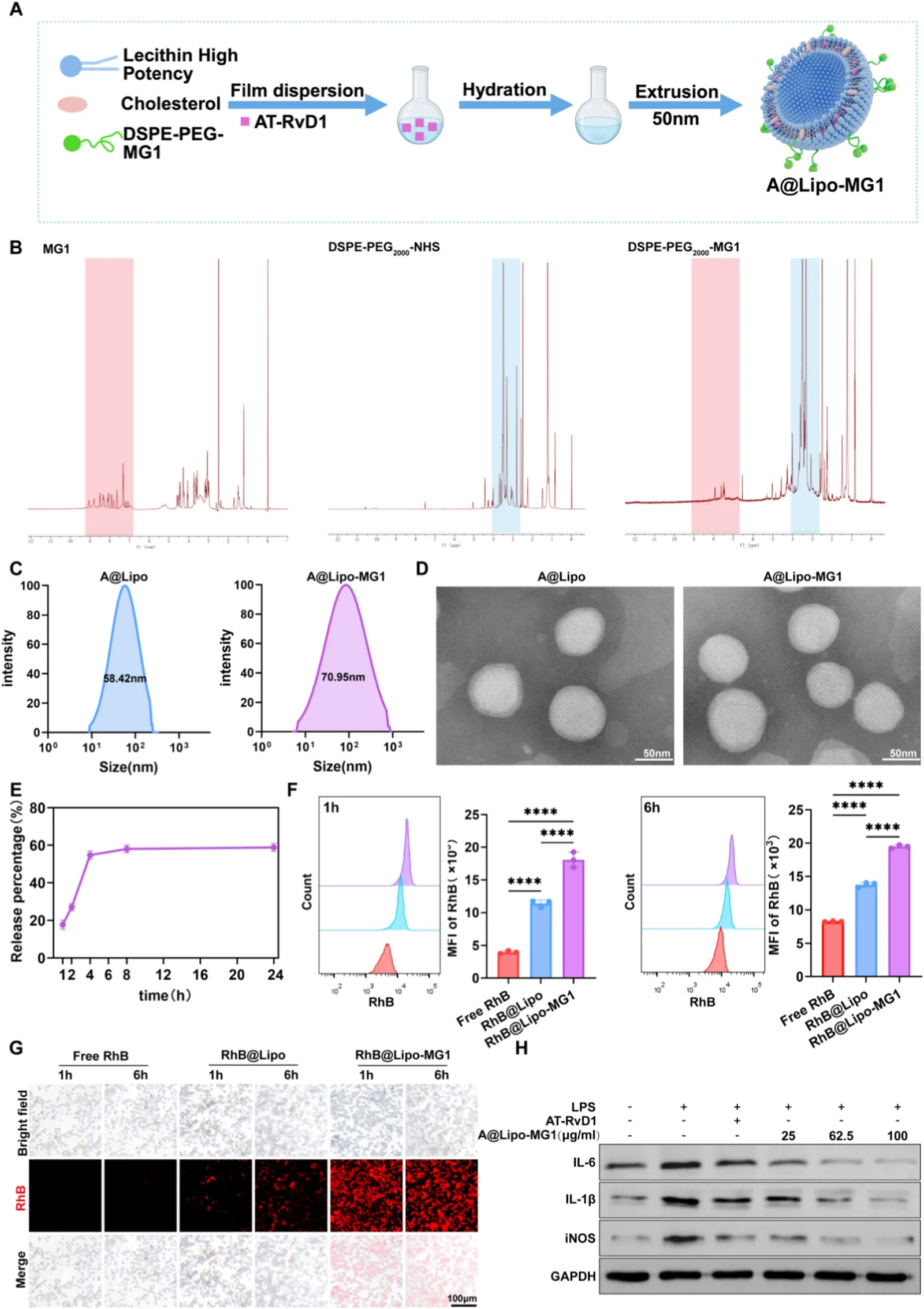
**Preparation, characterization, and in vitro uptake of A@Lipo-MG1. (A)** Schematic illustration of A@Lipo-MG1 nanoparticle preparation. Created in https://BioRender.com**(B)**^1^H NMR spectra of MG1, DSPE-PEG_2000_-NHS, and DSPE-PEG_2000_-MG1. **(C)** Particle size distribution of A@Lipo and A@Lipo-MG1 nanoparticles. **(D)** TEM images of A@Lipo and A@Lipo-MG1. Scale bar = 50 nm. **(E)** In vitro cumulative release profile of AT-RvD1 from A@Lipo-MG1 over 24 h, quantified by LC-MS. **(F)** Flow cytometric analysis of BV2 cell uptake of RhB@Lipo and RhB@Lipo-MG1. **(G)** Representative fluorescence microscopy images showing BV2 cell uptake of RhB@Lipo and RhB@Lipo-MG1. Scale bar = 100 μm. **(H)** IL-6, IL-1β, and iNOS protein expression in BV2 cells treated with increasing concentrations of A@Lipo-MG1 (25, 62.5, and 100 μg/mL) by western blot analysis. Data are presented as mean ± SD (n = 3 per group). *P < 0.05, **P < 0.01, ***P < 0.001, ****P < 0.0001 represent statistically significant differences between groups.

In vitro, we first assessed the cytotoxicity of A@Lipo-MG1. Compared with free AT-RvD1, A@Lipo-MG1 had no significant cytotoxicity (Fig. S5A), indicating excellent cellular biocompatibility of the liposome formulation. To assess cellular uptake efficiency, liposomes were labeled with Rhodamine B (RhB) and incubated with BV2 cells. Flow cytometry analysis revealed significantly higher intracellular uptake of RhB@Lipo-MG1 than RhB@Lipo in BV2 cells (Fig. 6F). Consistently, fluorescence microscopy showed stronger intracellular red fluorescence in the RhB@Lipo-MG1 group (Fig. 6G), indicating that MG1 modification enhanced microglial uptake of liposomes. We further treated LPS-stimulated BV2 cells with A@Lipo-MG1 at 25, 62.5, and 100 μg/mL, and Western blot analysis demonstrated that A@Lipo-MG1 significantly attenuated the inflammatory response in a concentration-dependent manner (Fig. 6H, Fig. S5B). To further investigate the BSCB penetration capacity of MG1-modified liposomes, we established an in vitro Transwell-based BSCB model (Fig. 7A). RhB-labeled liposomes without AT-RvD1 loading were first used to evaluate trans-BSCB transport. Compared with RhB@Lipo, RhB@Lipo-MG1 showed enhanced transport across the BSCB model, whereas pre-incubation with free MG1 for 1 h followed by medium replacement attenuated this enhancement (Fig. 7B). In a separate functional experiment, AT-RvD1-loaded liposomes without RhB labeling were applied to assess the biological effects after trans-BSCB transport. Western blot analysis of BV2 cells collected from the lower chamber showed that A@Lipo-MG1 more effectively reduced the expression of inflammatory proteins, while MG1 pre-incubation weakened this effect, further supporting the improved delivery and anti-inflammatory effects of AT-RvD1 associated with MG1 modification (Fig. 7C and Fig. S5C).

**Fig. 7:**
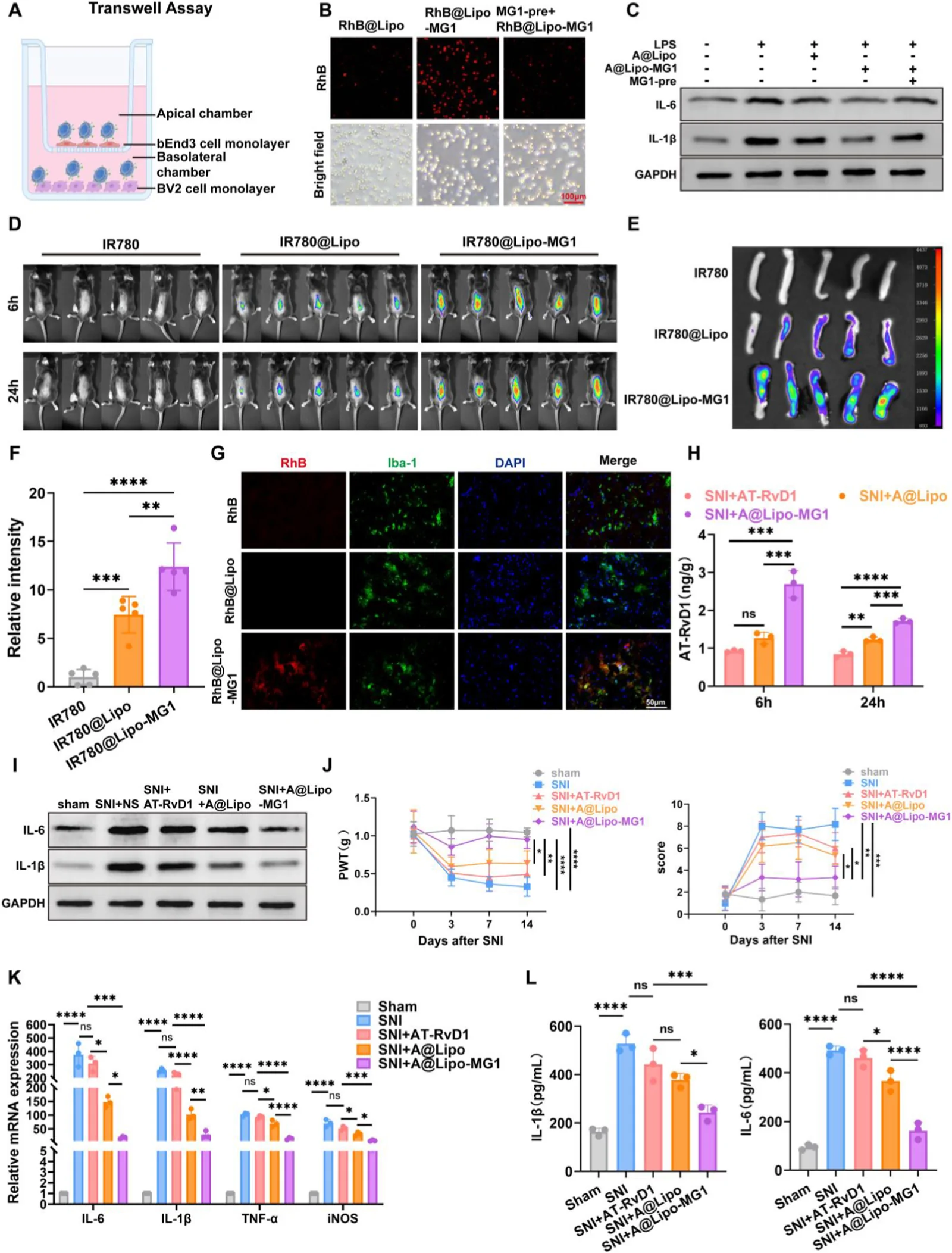
**In vivo uptake and therapeutic evaluation of A@Lipo-MG1. (A)** Schematic illustration of the Transwell-based BSCB penetration assay for A@Lipo-MG1. Created in https://BioRender.com. **(B)** Fluorescence images of BV2 cells in the lower chamber after treatment with RhB@Lipo, RhB@Lipo-MG1, or MG1-pre + RhB@Lipo-MG1. Scale bar = 100 μm. **(C)** Western blot analysis of IL-6 and IL-1β expression in BV2 cells from the lower chamber. **(D)** In vivo fluorescence imaging showing the biodistribution of IR780@Lipo and IR780@Lipo-MG1 in SNI mice (n = 5). **(E)** Fluorescence images of IR780 in excised spinal cords, with corresponding quantification of fluorescence intensity shown in **(F)**. **(G)** Representative immunofluorescence images of L4-L6 spinal dorsal horn microglia (green) showing RhB (red) uptake after RhB@Lipo or RhB@Lipo-MG1 treatment. Scale bar = 50 μm. **(H)** LC-MS/MS quantification of AT-RvD1 concentrations (ng/g tissue) in spinal cords after intravenous administration of free AT-RvD1, A@Lipo, or A@Lipo-MG1. **(I)** IL-6 and IL-1β protein levels in spinal cord tissues from different groups by western blot analysis. **(J)** Behavioral assessment of SNI mice, including PWT (g) and acetone test scores. (n = 6). **(K)** The mRNA transcript levels of IL-6, IL-1β, TNF-α, and iNOS in L4-L6 spinal cord segments of SNI mice treated with free AT-RvD1, A@Lipo or A@Lipo-MG1 by qRT-PCR. **(L)** ELISA analysis of IL-6 and IL-1β protein levels in L4-L6 spinal cord segments. Data are presented as mean ± SD (n = 3 per group). *P < 0.05, **P < 0.01, ***P < 0.001, ****P < 0.0001 represent statistically significant differences between groups. (For interpretation of the references to colour in this figure legend, the reader is referred to the Web version of this article.)

### 3.7 A@Lipo-MG1 efficiently targets spinal microglia and reduces inflammatory responses in vivo

To verify the spinal cord-targeting capacity of the liposomal carriers in vivo, free IR780, IR780@Lipo, and IR780@Lipo-MG1 were intravenously administered to mice, and fluorescence distribution was assessed at 6 h and 24 h using a small-animal in vivo imaging system. Free IR780 exhibited negligible fluorescence in the spinal cord at both time points, whereas IR780@Lipo showed relatively weak fluorescence. In contrast, IR780@Lipo-MG1 exhibited markedly enhanced fluorescence at both 6 h and 24 h (Fig. 7D). Ex vivo fluorescence imaging of harvested spinal cords at 24 h further confirmed the strongest fluorescence intensity in the IR780@Lipo-MG1 group (Fig. 7E and F), indicating enhanced spinal cord accumulation of the MG1-modified liposomal carrier. Immunofluorescence staining of L4-L5 spinal cord frozen sections showed that intravenous administration of RhB@Lipo-MG1 resulted in pronounced accumulation of RhB-labeled nanoparticles in Iba1+ microglia (Fig. 7G), verifying that MG1 modification substantially improves the targeting specificity of liposomes to spinal microglia. To further verify the delivery of AT-RvD1 to the spinal cord, its absolute concentration in spinal cord tissue was quantified by LC-MS/MS at 6 h and 24 h after intravenous administration in SNI mice (Fig. 7H). At 6 h, A@Lipo showed a modest but non-significant increase in spinal cord AT-RvD1 levels compared with free AT-RvD1. At 24 h, AT-RvD1 levels were higher in the A@Lipo group than in the free AT-RvD1 group. Notably, A@Lipo-MG1 significantly enhanced spinal cord AT-RvD1 accumulation and exhibited the highest levels at both time points. Additionally, the lower AT-RvD1 levels observed at 24 h may be attributable to time-dependent drug degradation and clearance. Collectively, these results demonstrate that A@Lipo-MG1 enhances the spinal cord delivery of AT-RvD1. Western blot analysis validated the anti-inflammatory efficacy of A@Lipo-MG1 (Fig. 7I, Fig. S5D). Consistent with these molecular findings, behavioral assessments demonstrated that intravenous administration of free AT-RvD1 at an equivalent dose produced only a minimal analgesic effect, whereas A@Lipo-MG1 significantly alleviated mechanical hypersensitivity in SNI mice (Fig. 7J). To further assess its anti-inflammatory effects in vivo, qRT-PCR analysis of harvested L4-L6 spinal cord segments showed that A@Lipo-MG1 significantly downregulated mRNA transcript levels of IL-6, IL-1β and TNF-α cytokines (Fig. 7K), consistent with the ELISA results of reduced IL-6 and IL-1β secretion (Fig. 7L). Collectively, A@Lipo-MG1 enables targeted delivery of AT-RvD1 to spinal microglia via intravenous injection, markedly increasing AT-RvD1 accumulation at the spinal cord and thereby enhancing its anti-inflammatory and analgesic function.

For biocompatibility assessment, serum liver and kidney function tests showed that none of the treatments caused abnormal changes in hepatic or renal function biomarkers (Fig. S5E). H&E staining of major organs (heart, liver, spleen, lung and kidney) further showed no obvious structural lesions or inflammatory infiltration after liposome administration (Fig. S5F), confirming the excellent in vivo safety of A@Lipo-MG1. In summary, A@Lipo-MG1 exhibits favorable biocompatibility both in vitro and in vivo, high targeting specificity to spinal microglia, and potent anti-inflammatory and analgesic efficacy, representing a promising targeted delivery platform for neuroinflammation intervention with substantial clinical translation potential.

## 4 Discussion

In this study, we demonstrated that AT-RvD1 inhibits Angptl4 expression, thereby weakening the protective effect of Angptl4 on Suclg1, which in turn promotes the ubiquitination of Suclg1 and its degradation via the proteasome pathway. This process affects the propionate levels in the spinal microenvironment, thereby facilitating a microglial phenotypic shift from a pro-inflammatory toward an anti-inflammatory and pro-repair state, alleviating neuroinflammation and improving pain hypersensitivity. In conclusion, this study provides evidence that AT-RvD1 improves spinal neuroinflammation and pain hypersensitivity induced by the SNI model by regulating the ANGPTL4-SUCLG1-propionate axis. Furthermore, we engineered A@Lipo-MG1 that enables minimally invasive, targeted delivery of AT-RvD1 to spinal microglia via intravenous administration, significantly enhancing the anti-inflammatory and analgesic efficacy of AT-RvD1 with excellent biocompatibility.

It is well established that the initiation and maintenance of NP are tightly linked to neuroinflammatory cascades in the spinal cord, where microglia serve as the principal innate immune effector cells, and the balance between pro-inflammatory and anti-inflammatory/pro-repair states functions as a critical hub connecting nerve injury, inflammation, and pain sensitization. Therefore, targeting microglial polarization has emerged as a promising strategy for analgesic development [8,42,43]. AT-RvD1, as a member of SPMs, participates in the active resolution of inflammation. Previous studies have shown that SPMs play an important role in regulating neuroinflammation, significantly suppressing inflammatory responses and promoting the restoration of tissue homeostasis [44]. Further evidence has demonstrated that AT-RvD1 exerts significant anti-inflammatory and pro-reparative effects [45,46]. In agreement with earlier reports emphasizing the fundamental involvement of microglial polarization in NP progression, our results showed that SNI induced pronounced microglial activation and a shift toward a pro-inflammatory state in the spinal dorsal horn, which was effectively reversed by AT-RvD1 treatment. Our work provides direct evidence that AT-RvD1 modulates dysregulated microglial inflammatory responses to alleviate NP, expanding the functional understanding of SPMs in pain management.

To identify the molecular target mediating the regulatory effects of AT-RvD1, we screened and validated Angptl4 as a key downstream effector of AT-RvD1 in microglia. Angptl4 plays a complex dual contribution in inflammation responses, with its function varying with dependence on the particular disease setting, cell type, and stage of pathology. For example, Angptl4 can promote keratinocyte proliferation and inflammatory responses in psoriasis, whereas during wound healing, it coordinates neutrophil clearance and the resolution of inflammation [47,48]. In neuroinflammation, Angptl4 also plays a dual role. It exhibits pro-inflammatory effects in Alzheimer's disease models, whereas in Parkinson's disease models, it can suppress endoplasmic reticulum stress [22,49]. These findings indicate that Angptl4 exerts complex regulatory functions in inflammation. According to our study, Angptl4 expression was significantly increased in the spinal cord of SNI model mice. Overexpression of Angptl4 enhanced microglial inflammation and promoted a shift toward a pro-inflammatory state, suggesting a pro-inflammatory role of Angptl4 in microglia. Treatment with AT-RvD1 markedly downregulated Angptl4 expression, whereas overexpression of Angptl4 attenuated the anti-inflammatory efficacy of AT-RvD1. Taken together, these findings indicate that AT-RvD1 regulates microglial inflammatory responses and alleviates pain hypersensitivity by downregulating Angptl4.

We further delineated the downstream signaling cascade of the AT-RvD1/ANGPTL4 axis. Proteomic profiling and enrichment analysis revealed that AT-RvD1 administration substantially decreased the expression of key enzymes involved in the propionate metabolism pathway, including Suclg1. To explore the underlying mechanism, IP-MS in BV2 cells identified Suclg1 as a potential interacting partner of Angptl4. As a key enzyme in both the tricarboxylic acid cycle and propionate metabolism, Suclg1 has been reported that inhibition of Suclg1 expression in macrophages promotes the accumulation of the anti-inflammatory metabolite itaconate, thereby exerting anti-inflammatory effects [41]. In this study, we demonstrated for the first time that Angptl4 directly interacts with Suclg1 and inhibits its degradation via the ubiquitin-proteasome pathway, whereas AT-RvD1 promotes Suclg1 degradation by suppressing Angptl4, thereby modulating microglial inflammatory responses.

Previous studies have indicated that propionate exhibits predominantly anti-inflammatory properties in various inflammatory models [50,51]. In the present study, spinal propionate levels were markedly decreased in SNI model mice, whereas AT-RvD1 treatment significantly increased propionate levels. In addition, results from both in vivo and in vitro studies indicated that propionate attenuated microglial inflammatory responses and promoted a shift toward an anti-inflammatory and pro-repair state, leading to reduced pain hypersensitivity, in agreement with previous findings. Taken together, these findings suggest that AT-RvD1 may ameliorate SNI-induced spinal neuroinflammation and pain hypersensitivity by modulating the ANGPTL4-SUCLG1-propionate axis, thus facilitating the transition of microglia from a pro-inflammatory toward an anti-inflammatory and pro-repair state.

Despite the promising therapeutic potential of AT-RvD1, its clinical application is restricted due to rapid degradation and poor stability in vivo, as well as the invasive nature of intrathecal administration, which is unsuitable for long-term repeated use. To overcome these barriers, liposomal delivery systems have been broadly applied as a result of their good biocompatibility and strong drug-loading capacity, and ability to enhance drug stability, improve in vivo distribution, and enable targeted delivery [[52], [53], [54]]. In this study, we therefore developed an MG1 peptide-modified liposomal system encapsulating AT-RvD1 (A@Lipo-MG1) to address these limitations. The prepared A@Lipo-MG1 displayed a uniform particle size, regular spherical morphology, and excellent colloidal stability. In vitro studies showed that MG1 modification markedly enhanced liposomal uptake by activated microglia, and A@Lipo-MG1 exerted a dose-dependent anti-inflammatory effect in LPS-stimulated cells. Importantly, in vivo imaging and histological analyses confirmed that intravenous administration enabled efficient targeting of AT-RvD1 to spinal microglia, resulting in greater accumulation at lesion sites compared with free AT-RvD1 and non-targeted liposomes. Functionally, A@Lipo-MG1 exhibited more potent anti-inflammatory and analgesic effects than free AT-RvD1 in the SNI mouse model, with no obvious hepatotoxicity, nephrotoxicity or organic pathological damage observed in vivo. Compared with conventional intrathecal administration, this intravenous targeted delivery strategy is minimally invasive and suitable for long-term repeated administration, providing a feasible solution for the clinical translation of AT-RvD1.

This study also has several limitations. First, we did not further investigate how Angptl4 inhibits Suclg1 degradation via the ubiquitin-proteasome pathway. Second, the binding regions and sites between Angptl4 and Suclg1 were not explored. Third, only male mice were used in the present study. Given that sex-related differences may influence neuroinflammatory responses and pain sensitivity, the generalizability of our findings to females remains to be determined. Future studies including both sexes are warranted to further validate these findings. In addition, the assessment of microglial states in this study was primarily based on a limited set of phenotypic markers, which may not fully capture the heterogeneity and functional complexity of microglial activation. Finally, the long-term pharmacokinetics and safety of A@Lipo-MG1 in larger animal models remain to be evaluated.

## 5 Conclusion

This study demonstrates that AT-RvD1 alleviates nociceptive hypersensitivity in neuropathic pain by inhibiting Angptl4 expression, reducing Suclg1 stability, and regulating downstream inflammatory responses, thereby increasing spinal propionate levels and facilitating the transition of microglia toward an anti-inflammatory state. Furthermore, the A@Lipo-MG1 delivery platform developed in this study enables precise delivery of AT-RvD1 to spinal microglia, providing a potentially translatable therapeutic strategy for neuropathic pain. Collectively, this study not only elucidates the key regulatory network of AT-RvD1 but also offers new therapeutic targets and insights for the intervention of neuropathic pain.

## CRediT authorship contribution statement

**Kaili Wu:** Writing – original draft, Visualization, Validation, Methodology, Conceptualization. **Chao Gao:** Validation, Methodology, Conceptualization. **Shunli Fu:** Validation, Methodology, Conceptualization. **Yan Li:** Data curation. **Zhaohui Li:** Validation, Data curation. **Xinyi Han:** Validation, Investigation. **Yun Zhang:** Investigation. **Yingqi Zhang:** Validation. **Yihao Wang:** Writing – review & editing, Supervision, Resources, Project administration, Funding acquisition.

## Ethics approval

All experimental animal procedures were followed and approved by the Experimental Animal Care and Use Committee of the Institute of Qingdao University(AHQU-MAL20251125WKL).

## Funding

This work was supported by the 10.13039/501100007129Shandong Provincial Natural Science Foundation [ZR2022MH110, ZR2026MS1366].

## Declaration of competing interest

The authors declare that they have no known competing financial interests or personal relationships that could have appeared to influence the work reported in this paper.

## Data Availability

Data will be made available on request.
