# Supplementary material for: AT-RvD1 liposomes effectively accumulate in spinal cord and reprogram microglia via ANGPTL4-SUCLG1-propionate axis for treating neuropathic pain
Source: Mater Today Bio. 2026 Oct 5;41:103726. doi: 10.1016/j.mtbio.2026.103726 (PMC13678001; doi:10.1016/j.mtbio.2026.103726)
Supplement: Supplementary file 1 [file mmc1.docx]

AT-RvD1 Liposomes Effectively Accumulate in Spinal Cord and Reprogram Microglia via ANGPTL4–SUCLG1–Propionate Axis for Treating Neuropathic Pain

**Fig. S1.** Validation of the therapeutic effects of AT-RvD1. (A) Densitometric analysis of protein expression from the Western blot shown in Figure 2C. (B) Quantitative analysis of fluorescence intensity for Iba1, IL-6, IL-1β, CD86, and CD206 staining. (C) Cell viability of BV2 cells treated with different concentrations of AT-RvD1, assessed by CCK-8 assay. (D, E) The anti-inflammatory effects of AT-RvD1 in a dose-dependent manner were validated by qRT-PCR (D) and Western blot (E). (F) Densitometric analysis of protein expression from the Western blot shown in Figure 2E. (G) IL-6 expression in BV2 cells was measured by ELISA. (H) Quantification of CD86 and CD206 fluorescence intensity from confocal imaging. Data are presented as mean ± SD (n = 3 per group). *P < 0.05, **P < 0.01, ***P < 0.001, ****P < 0.0001 indicate statistically significant differences.

**Fig. S2.** Role of Angptl4 in neuropathic pain and BV2 cells. (A) qRT-PCR analysis of candidate genes, including Angptl4, in BV2 cells treated with LPS or LPS combined with AT-RvD1. (B) Densitometric analysis of protein expression from the Western blots shown in Figure 3C. (C) Quantification of fluorescence intensity of Angptl4 immunostaining in BV2 cells. (D) Densitometric analysis of protein expression from the Western blots shown in Figure 3D. (E) Quantification of fluorescence intensity of Angptl4 immunostaining in spinal cord tissues. (F) Knockdown efficiency of Angptl4 in BV2 cells transfected with siAngptl4-1/-2 or siNC, as confirmed by qRT-PCR. (G) Densitometric analysis of protein expression from the Western blot shown in Figure 3G. (H) Overexpression efficiency of Angptl4 in BV2 cells transfected with OE-Angptl4 or vector, as validated by qRT-PCR. (I–K) Densitometric analysis of protein expression from the Western blots shown in Figure 3H, I, and K. (L) Quantification of CD86 and CD206 fluorescence intensity from confocal imaging. Data are presented as mean ± SD (n = 3 per group). *P < 0.05, **P < 0.01, ***P < 0.001, ****P < 0.0001 indicate statistically significant differences.

**Fig. S3.** Validation of Angptl4-mediated regulation of Suclg1. (A) Densitometric analysis of protein expression from the Western blots shown in Figure 4J. (B) Knockdown efficiency of Suclg1 in BV2 cells transfected with siSuclg1-1/-2 or siNC, as confirmed by qRT-PCR. (C) Densitometric analysis of protein expression from the Western blots shown in Figure 4K. Data are presented as mean ± SD (n = 3 per group). *P < 0.05, **P < 0.01, ***P < 0.001, ****P < 0.0001 indicate statistically significant differences.

**Fig. S4.** Validation of Angptl4-mediated regulation of Suclg1 degradation and the synergistic anti-inflammatory effects of NaP and AT-RvD1. (A, B) Densitometric analysis of protein expression from the Western blots shown in Figure 5A，B. (C, D) qRT-PCR analysis of Suclg1 mRNA levels in BV2 cells with Angptl4 overexpression or knockdown. (E,F) Densitometric analysis of protein expression from the Western blots shown in Figure 5I, J. (G) PWT(g) and acetone test scores in SNI mice treated with AT-RvD1, NaP, or their combination (n = 6). (H)Densitometric analysis of protein expression from the Western blots shown in Figure 5L. Data are presented as mean ± SD (n = 3 per group). *P < 0.05, **P < 0.01, ***P < 0.001, ****P < 0.0001 indicate statistically significant differences.

**Fig. S5.** Evaluation of the therapeutic effects of A@Lipo-MG1. (A) Cell viability of BV2 cells treated with A@Lipo or A@Lipo-MG1, assessed by CCK-8 assay. (B) Densitometric analysis of protein expression from the Western blots shown in Figure 6H. (C,D) Densitometric analysis of protein expression from the Western blots shown in Figure 7C and Figure 7I. (E)Serum biochemical analysis 24 h after injection of free AT-RvD1, A@Lipo or A@Lipo-MG1, including ALT, AST, TBIL, Alb, BUN, and Cr. (F) Histological analysis was performed by H&E staining on major organs (heart, liver, spleen, lung, and kidney) 24 h following administration of free AT-RvD1, A@Lipo or A@Lipo-MG1. Scale bar = 100 μm. Data are presented as mean ± SD (n = 3 per group). *P < 0.05, **P < 0.01, ***P < 0.001, ****P < 0.0001 indicate statistically significant differences.

**Supplementary Table 1.** The primer sequences of qRT-PCR

| Gene Name | Primers |
| --- | --- |
| β-actin | Forward：TATGCTCTCCCTCACGCCATCC  Reverse：GTCACGCACGATTTCCCTCTCAG |
| IL-1β | Forward：TCGCAGCAGCACATCAACAAGAG  Reverse：AGGTCCACGGGAAAGACACAGG |
| IL-6 | Forward：TCTGGAGCCCACCAAGAACGATAG  Reverse：GTCACCAGCATCAGTCCCAAGAAG |
| iNOS | Forward：ACTCAGCCAAGCCCTCACCTAC  Reverse：TCCAATCTCTGCCTATCCGTCTCG |
| Arg-1 | Forward：AACCTTGGCTTGCTTCGGAACTC  Reverse：GTTCTGTCTGCTTTGCTGTGATGC |
| TNF-α | Forward：CACGCTCTTCTGTCTACTGAACTTC  Reverse：CTTGGTGGTTTGTGAGTGTGAGG |
| Angptl4 | Forward：GAACGGCTCTGTGGACTTCAACC  Reverse: TGGCTTCCTCGGTTCCCTGTG |
| Suclg1 | Forward：GGATACGACACGGGTCTTACA  Reverse：GTGGTTCCTCCCACGAGTTTG |
| Hsf4 | Forward：TCAACTCCTTGACCGTCAGC  Reverse: CTGCCCCTGAAACTCTGGAG |
| Scn10a | Forward：TCACTCGGGGCTTTTCTTCC  Reverse:CCTGCTGCAAAGTGAACACC |
| Ahsa1 | Forward：TCCATGCACAGCTCTGTCTC  Reverse:CAACGTCACCGGGGAGTTTA |
| Nell1 | Forward：GTCACAGTTGTGTTGTCCGC  Reverse:CTTGCCGGGGTTATATCGCT |
| Cadm1 | Forward：CCAATGGTCCCCTCTTCACC  Reverse:AACATGCCGTACTGTCTGGG |
| Gls2 | Forward：GCACAATGTTGCTGCTCACA  Reverse:AGTTCACCACGGCTCTGAAG |
| Trim2 | Forward：CGACTCGGAAGATCAGGTCG  Reverse:AAGGCTGTGAAGAGACCTGC |
| Luzp1 | Forward：CCATTCCCATGTTGGAGGCT  Reverse:CGGAACAGGGAGTTTGCTCT |
| Usp8 | Forward：GCTTGTTTTCCCGGTTGACC  Reverse:ACAGGGAGCCATCGAAACTG |
| Ass1 | Forward：GGGGTTTTCCAGGATCCCAG  Reverse:TAAGGTCATCGCTCCCTGGA |
| Nrxn1 | Forward：GGCTGTCGACTCAGTTGTCA  Reverse:AGCAGAGAACGATGCCAACA |
| Baiap2l1 | Forward：CCCGCTTTCTTGTCTGAGGT  Reverse:GTCTGTCCCTACACCAAGCC |
| Asb8 | Forward：GCATTGACCTCTGCTCCCTT  Reverse:ATCAGAGGGGGAGCAGATGT |
| Fn1 | Forward：AGGGGATCCAGGCTTCTCAT  Reverse:CGTGATCATCGATGCCTCCA |
| Vgf | Forward：CCTGGAATTGGGAAGGGACC  Reverse:TGCTAAGCGCCAACAAGAGA |
| Lyz2 | Forward：GGGATCTCTCACCACCCTCT  Reverse:GCAAAACCCCAAGAGCTGTG |
| Arpc1b | Forward：CGTGCACCTTGTTCCACTTG  Reverse:ACCGTACACAGATCGCCATC |
| Litaf | Forward：CACAGACTGCCACAGGAGAG  Reverse:AGATGTGCTGTCCTTCCTGC |
| Timp1 | Forward：TCTGGTAGTCCTCAGAGCCC  Reverse:TGTGCACAGTGTTTCCCTGT |
| Jun | Forward：CACCTGTTCCCTGAGCATGT  Reverse:CAAGTGCCGGAAAAGGAAGC |
| Prdx4 | Forward：GGTGCTGGCTTGGAGATCTT  Reverse:CTACGCTGGTGGACAAGTGT |
| Hdac11 | Forward：TGGCCTCTTTAGCAAAGCGA  Reverse:GCGAGACTTCATGGGTGACA |
| Marcks | Forward：TCCTGCCCATTTGCTTTGGA  Reverse:GTGCCCAGTTCTCCAAGACC |
| Ehbp1 | Forward：GCTGAACTGCTTTGTTGGGG  Reverse:ACCATGTGCTTCTCTGAGGC |
| Tpm4 | Forward：AGTTCAGATACCTCCGCCCT  Reverse:GGAGATCCTGGAGATGCAGC |
| Sept2 | Forward：TGCATGTGGGTGATGAGCAT  Reverse:GCAAGAAGGTTAGAGGCCGT |
| Xbp1 | Forward：CCTCCCAGGAGTGGTCTGTA  Reverse:GAGCTGGGCATCTCAAACCT |
| Prkd3 | Forward：TGCTGCTCCTCCTCTTTGTG  Reverse:CGACTGATCTCGACGTGGAG |
| COX2 | Forward：ACTCTGTTGTGCTCCCGAAG  Reverse:GAACCTGCAGTTTGCTGTGG |
